# Comparative transcriptome analysis reveals key pathways and genes involved in trichome development in tea plant (*Camellia sinensis*)

**DOI:** 10.3389/fpls.2022.997778

**Published:** 2022-09-23

**Authors:** Lan Chen, Na Tian, Mengqing Hu, Devinder Sandhu, Qifang Jin, Meiyi Gu, Xiangqin Zhang, Ying Peng, Jiali Zhang, Zhenyan Chen, Guizhi Liu, Mengdi Huang, Jianan Huang, Zhonghua Liu, Shuoqian Liu

**Affiliations:** ^1^Department of Tea Science, College of Horticulture, Hunan Agricultural University, Changsha, China; ^2^Key Laboratory of Tea Science of Ministry of Education, Hunan Agricultural University, Changsha, China; ^3^Xiangxi Academy of Agricultural Sciences, Jishou, China; ^4^United States Salinity Laboratory, United States Department of Agriculture, Agricultural Research Service, Riverside, CA, United States

**Keywords:** *Camellia sinensis*, tea leaf, trichome, transcriptome, differentially expressed genes

## Abstract

Trichomes, which develop from epidermal cells, are considered one of the important characteristics of the tea plant [*Camellia sinensis* (L.) O. Kuntze]. Many nutritional and metabolomic studies have indicated the important contributions of trichomes to tea products quality. However, understanding the regulation of trichome formation at the molecular level remains elusive in tea plants. Herein, we present a genome-wide comparative transcriptome analysis between the hairless Chuyeqi (CYQ) with fewer trichomes and the hairy Budiaomao (BDM) with more trichomes tea plant genotypes, toward the identification of biological processes and functional gene activities that occur during trichome development. In the present study, trichomes in both cultivars CYQ and BDM were unicellular, unbranched, straight, and soft-structured. The density of trichomes was the highest in the bud and tender leaf periods. Further, using the high-throughput sequencing method, we identified 48,856 unigenes, of which 31,574 were differentially expressed. In an analysis of 208 differentially expressed genes (DEGs) encoding transcription factors (TFs), five may involve in trichome development. In addition, on the basis of the Gene Ontology (GO) annotation and the weighted gene co-expression network analysis (WGCNA) results, we screened several DEGs that may contribute to trichome growth, including 66 DEGs related to plant resistance genes (PRGs), 172 DEGs related to cell wall biosynthesis pathway, 29 DEGs related to cell cycle pathway, and 45 DEGs related to cytoskeleton biosynthesis. Collectively, this study provided high-quality RNA-seq information to improve our understanding of the molecular regulatory mechanism of trichome development and lay a foundation for additional trichome studies in tea plants.

## Introduction

Trichomes are epidermal outgrowths on the leaves, stems, flowers, and inflorescence stems of most terrestrial plants (Zhang et al., 2021). Based on the morphology, trichomes, can generally be divided into single-cellular or multicellular, branched or branchless, and glandular or glandless (Yang and Ye, 2013). Trichomes are thought to be a crucial protective barrier against natural hazards, such as protecting plants from herbivores and pathogens (fungi and bacteria) attacks, preventing ultraviolet (UV) radiation and high light damage, and providing drought and cold resistance (Kariyat et al., 2018). Additionally, trichomes in the cotton seed coat (also referred to as cotton fiber), are an important resource and raw material for the fiber and textile industry (Ma et al., 2016). The glandular trichomes of *Artemisia annua* can produce artemisinin, a well-known antimalarial drug (Zhou et al., 2020b). Thus, trichomes are economically important for plant breeding programs.

The morphogenesis and molecular regulation mechanism of trichomes have been well studied in many plant species, such as *Arabidopsis* (Doroshkov et al., 2019), cucumber (Liu et al., 2016), tobacco (Harada et al., 2010), tomato (Chang et al., 2019), cotton (Ma et al., 2016), and so on. The trichome development process is coordinated and regulated by various factors, including environment, phytohormones, regulatory genes (functional genes and transcription factors), and non-coding RNAs. Previous studies demonstrated that a series of transcription factors (TFs) play vital roles in the regulation of initiation, growth, and development of plant trichomes (Doroshkov et al., 2019; Fambrini and Pugliesi, 2019; Wang et al., 2021; Zhang et al., 2021). Among them, positive regulatory TFs, including WD40 TF family protein TRANSPARENT TESTA GLABRA1 (TTG1), bHLH TF family proteins MYC-1, GLABRA3 (GL3), ENHANCER OF GLABRA3 (EGL3), and TRANSPARENT TESTA 8 (TT8), MYB TF family proteins GLABRA 1 (GL1), MYB75, MYB23, and MYB5 (Wang et al., 2021). These TFs are functionally redundant and form an MYB-bHLH-WD40 (MBW) complex to bind to the promoter of an HB TF protein GLABRA2 (GL2) (Wang and Chen, 2008). Negative regulatory TFs, including MYB TF family protein ENHANCER OF TRY AND CPC 1 (ETC1), ENHANCER OF TRY AND CPC 2 (ETC2), TRIPTY CHON (TRY), and CAPRICE (CPC) (Szymanski et al., 2000). ETC1 and ETC2 act as enhancers of TRY and CPC to facilitate the movement of TRY in cells and inhibit MBW complex formation (Esch et al., 2003). Other TFs such as those encoding the MYB TF protein NOECK (NOK) (Glover et al., 1998; Ewas et al., 2016), HB TF protein MERISTEM L1 (ATML1) (Abe et al., 2003; Nakamura et al., 2006; Takada et al., 2013), SPL TF proteins SPL6 and SPL12 (Yu et al., 2010; Bhogale et al., 2014; Aung et al., 2015) have been shown to regulate trichome development in many plant species.

In addition, cell walls are vital for trichomes in supporting morphological variations and defensive functions. Generally, plant trichome cell walls contain pectin, cellulose, lignin, and mannose-containing polysaccharides (MCPs), and are covered with cuticular wax (Marks et al., 2008). Previous studies revealed that the functional genes involved in cell wall function, biosynthesis, and structure were expressed at high levels in plant trichomes (Jakoby et al., 2008; Marks et al., 2009). For example, some genes for cellulose and cuticular wax biosynthesis, such as *cellulose synthase A* (*CESA*), *glycosyl-phosphatidyl inositol-anchored* (*COBRA*), *trichome birefringence-like* (*TBL*), β*-ketoacyl-CoA synthase* (*KCS*), *hydroxyacyl-CoA dehydratase* (*HCD*), *enoyl-CoA reductase 1* (*CER1*), *mid-chain hydroxylase 1* (*MAH1*), *fatty-acyl CoA reductase* (*FAR*), *wax ester synthase 1* (*WSD1*), and *long-chain acyl-CoA synthases* (*LACS*) have been extensively studied in *Arabidopsis* trichomes and cotton fiber (Betancur et al., 2010; Bischoff et al., 2010; Xie et al., 2013; Niu et al., 2015; Busta et al., 2016; Hegebarth et al., 2016). Some genes involved in lignin biosynthesis, such as *phenylalanine ammonia-lyase* (*PAL*), *4-coumarate-CoA ligase* (*4CL*), *cinnamate-4-hydroxylase* (*C4H*), *cinnamoyl-CoA reductase* (*CCR*), *cinnamyl alcohol dehydrogenase* (*CAD*), *hydroxycinnamoyl-CoA transferase* (*HCT*), *caffeoyl-CoA O-methyltransferase* (*CCoAOMT*), *caffeic acid O methyltransferase* (*COMT*), *ferulate 5-hydroxylase* (*F5H*), *laccase* (*LAC*), and *peroxidase* (*PER*) were elucidated in cotton fiber and fruit prickle (also referred to as fruit trichomes) (Xu et al., 2011; Lu et al., 2020; Zhang et al., 2020). Moreover, some cell cycle regulation and cytoskeleton structure-related genes, such as *cyclin B* (*CycB*) (Gao et al., 2017), *cell division cycle* (*CDC*) (Park et al., 2008), *actin* (*ACT*) (Kandasamy et al., 2002; Li et al., 2005), *tubulin* (*TUB*) (Tatsuya et al., 2004; Abe and Hashimoto, 2010), *actin depolymerizing factor* (*ADF*) (Burgos-Rivera et al., 2008; Wang et al., 2019), *microtubule associated protein* (*MAP*) (Perrin et al., 2007; Zhu et al., 2018), *kinesin* (*KIS*) (Oppenheimer et al., 1997; Preuss et al., 2004; Lu et al., 2005), and *myosin* (*MYO*) (Ojangu et al., 2007, 2012) have been extensively studied in plant trichomes. Recently, increasing evidence has suggested that plant trichome development is regulated by a series of microRNAs (miRNAs) (Wang et al., 2021). Plant miRNAs are a class of small, endogenous, non-coding RNAs of 20-24 nucleotides in length with high sequence complementarity to their target mRNAs (Song et al., 2019). They attend to many aspects of cellular functions by modulating the expression levels of their target mRNAs at the post-transcriptional level (Fambrini and Pugliesi, 2019). Several miRNAs such as miR156 (Aung et al., 2015), miR157 (He et al., 2018), miR171 (Xue et al., 2014), miR319 (Fan et al., 2020), miR828 (Guan et al., 2014), and miR858 (Guan et al., 2014), etc., have been identified to regulate trichome differentiation and morphogenesis.

Tea [*Camellia sinensis* (L.) O. Kuntze] is one of the most popular non-alcoholic beverage crops worldwide, due to its rich flavors and numerous health benefits. The fresh and tender leaves used as tea processing materials are usually covered with an abundance of trichomes, also referred to as “Cháháo” in China (Li et al., 2020b). The presence of long and densely spaced trichomes on buds and tender leaves of tea plants is a crucial feature associated with enhanced plant defense against various environmental stresses through space hindrance and is also regarded as one of the most critical morphological markers for superior tea quality. Many nutritional and metabolomic studies have suggested the important contributions of trichomes to tea quality (Cao et al., 2020; Li et al., 2020a,b). Further, the density of trichomes in a tea plant is regulated by genetic parameters (Yue et al., 2018). Tea plants with a high density of trichomes or obvious glabrous leaves can be successfully selected by manual selective breeding techniques. Nevertheless, the molecular regulatory mechanisms underlying the trichome development process in tea plants warrant further study.

In the present study, we found that the tea plant trichomes belong to unicellular, unbranched, straight, and soft structured types, as observed by scanning electron microscopy (SEM). We further compared the transcriptome profiles between the hairless tea cultivar Chuyeqi (CYQ) and the hairy tea cultivar Budiaomao (BDM) from buds, first leaves, and fourth leaves. Our data indicated that differentially expressed genes (DEGs) related to trichome development were mainly enriched in Gene Ontology (GO) terms such as TFs, cytoskeleton structure, cell wall structure, cell cycle, and hormone regulation. Finally, we identified several possible candidate genes and pathways regulating trichome development by GO annotation and the weighted gene co-expression network analysis (WGCNA) results.

## Materials and methods

### Tea plant materials

In this study, two tea plant cultivars (*C. sinensis* cv. CYQ and BDM) were used as experimental materials. CYQ, is a national tea plant cultivar widely cultivated in tea-producing areas of China and has fewer trichomes in leaves. We selected CYQ sample from the tea plantation of Hunan Agricultural University (Changsha, Hunan, China, 28°10′ N, 113°05′E). BDM, is a newly discovered tea plant cultivar in Lijiawan village (Ningxiang city, Hunan province, China, 28°04′ N, 112°21′E). Buds (CYQ1 and BDM1), first leaves (CYQ2 and BDM2), and fourth leaves (CYQ3 and BDM3) from healthy tea plants were harvested in the spring (April 2019) from 8:30–11:00 a.m. A portion of fresh samples was collected in liquid nitrogen and kept at −80°C for transcriptome analysis. The remaining tea samples were used for SEM analysis. The above samples were displayed with three biological replicates for each cultivar.

### SEM analysis

For SEM analysis, tea leaves were cut into no more than 3 mm^2^ small pieces, and immediately fixed with electron microscopy fixative (Servicebio, Wuhan, China) for 12 h at 4°C. Afterward, the tea plant material was washed three times with 0.1 M phosphate-buffered saline solution (pH 7.4) for 15 min each. Then, the plant material was transferred into 1% OsO_4_ in 0.1 M phosphate-buffered saline solution for 1–2 h at room temperature, followed by three washes in 0.1 M phosphate-buffered saline solution for 15 min each. Tea plant samples were dehydrated with an ethanol dilution series (30, 50, 70, 80, 90, 95, 100, and 100%) for 15 min each time, and finally dehydrated with isoamyl acetate for 15 min. Subsequently, tea plant materials were dried overnight in a critical point dryer (Quorum K850, Quorum Technologies Ltd, Lewes, UK). After that, each specimen was gold coated in an ion sputtering apparatus (HITACHI MC1000, Tokyo, Japan) for about 30 s. The coated specimens were observed and photographed with an SEM (HITACHI SU8100, Tokyo, Japan). In addition, the number, length, and width of tea plant trichomes from each cultivar were calculated by Image-pro plus (v6.0) software (Media Cybernetics, Inc., Rockville, MD, USA).

### RNA extraction, library preparation, and transcriptome sequencing

Total RNA was extracted from fresh samples by using RNA plant Plus Reagent Kit (TIANGEN, Beijing, China) in accordance with the manufacturer's instructions. The quality, purity, concentration, and integrity of the total RNA were assessed separately using 1% agarose gel electrophoresis, NanoPhotometer spectrophotometer (IMPLEN, CA, USA), and Agilent 2100 bioanalyzer (Agilent Technologies, CA, USA). A total amount of 1 μg qualified RNA for each of the 18 samples (i.e., three biological replicates for the CYQ1, CYQ2, CYQ3, BDM1, BDM2, and BDM3 tea plant samples) was used to construct RNA sequencing libraries using the NEBNext^®^ UltraTM RNA Library Prep Kit for Illumina^®^ (NEB, Ipswich, MA, USA) following manufacturer's instructions. To preferentially select cDNA fragments with 250-300 bp length, the AMPure XP system (Beckman Coulter, Brea, CA, USA) was used to purify the library fragments. Then, 3 μL USER Enzyme (NEB, Ipswich, MA, USA) was used with size-selected, adaptor-ligated cDNA at 37 °C for 15 min followed by 5 min at 95°C before PCR assay. The PCR products were displayed with the AMPure XP system, and library quality was calculated with the Agilent Bioanalyzer 2100 system. Subsequently, the clustering of the index-coded samples was generated on a cBot Cluster Generation System using TruSeq PE Cluster Kit v3-cBot-HS (Illumina, San Diego, CA, USA) according to the vendor's recommendations. After cluster generation, each library was sequenced on an Illumina Novaseq platform at the Novogene Corporation (Novogene, Beijing, China). All sequencing data were deposited in the NCBI Sequence Read Archive (accession number PRJNA858236).

After removing adapter reads, ploy-N reads, and low-quality reads, the high-quality clean data were used for the Q20, Q30, and GC content determination. Then, the clean reads were uniquely aligned to the tea plant reference genome (*C. sinensis* cv. “Shuchazao”) (Wei et al., 2018) by using the HISAT (v2.0.5) software (Kim et al., 2015). The StringTie (v1.3.3) software (Pertea et al., 2015) was used to assemble the transcripts of each experiment separately. The gene expression levels were calculated by the fragments per kilobase per million reads (FPKM) method in Feature Counts (v1.5.0) software (Liao et al., 2014). The annotated transcripts of known mRNAs and novel mRNAs were designated as TEA_ID and novel_ID, respectively. For the DEGs analysis, the significantly differentially expressed genes in CYQ1, CYQ2, CYQ3, BDM1, BDM2, and BDM3 samples were calculated by the DESeq2 R package (v1.16.1) (Love et al., 2014), and the threshold corrected *p*-value for significance set as 0.05.

### Gene annotation, enrichment, and differential expression analysis

The unigenes were blasted against the GenBank Non-redundant, the Protein family (Pfam), the Swiss-Prot, the Karyotic Ortholog Groups (KOG), the GO database, and the Kyoto Encyclopedia of Genes and Genomes (KEGG) database, to obtain the annotation information. The DEGs were further conducted by mapping the GO and KEGG functional assignment and categorization. The methods for the GO and KEGG enrichment analyses were implemented by using the clusterProfiler R package (v3.4.4) (Yu et al., 2012). The overrepresentation of the GO terms and KEGG pathways were identified by calculating the false discovery rate (FDR) value. The FDR ≤ 0.05 was the threshold for significant enrichment.

### TFs analysis

The genes were annotated in the plant transcription factor database (PlantTFDB v4.0) (Jin et al., 2017) to determine whether they were TFs. The *p* ≤ 0.05 was set as the threshold value. The tea plant miRNA datasets were obtained from published studies (Guo et al., 2017; Jeyaraj et al., 2017a,b). The complementary sequences of identified TFs to miRNAs were searched using the psRNATarget (Dai et al., 2018) software. The expected value was set as 5.0, and the TF-miRNA interaction networks were visualized using the Cytoscape (v3.9.0) software (Shannon et al., 2003).

### Co-expression network analysis

The WGCNA was displayed by using the WGCNA R package (Langfelder and Horvath, 2008). The genes with FPKM > 1 and coefficient of variation (cv) value > 0.5 were retained to construct the co-expression networks. Further, the matrix of pairwise PCC between all unigene pairs was calculated. The matrix was converted to an adjacency matrix *via* raising the co-expression measure (0.5 + 0.5 × PCC). The power β set as 26 was the threshold value. Then, the resultant adjacency matrix was used to create the topological overlap matrix (TOM). The genes were hierarchically clustered through TOM similarity, and the dynamic hybrid tree cutting algorithm was used to cut the hierarchical clustering tree. For detecting modules, the minimum module size was set as 30, the minimum height for merging modules was set as 0.3, and the other parameters were set to default values. Each module was assessed by the first principal component of the scaled gene expression levels (module eigengene, ME). In addition, module membership (kME) was used to evaluate gene connectivity, and the genes (kME value > 0.7) were selected as the module members for further analysis. Moreover, based on PCC analysis, the three target traits (tea plant trichome number, length, and width) were used as phenotypic data for module-trait relationships. Gene significance was used to correlate trait data with the individual gene expression data. The co-expression networks of genes related to the specific trait (Top 200 unigene pairs) in each significant module were visualized using the Cytoscape (v3.9.0) software (Shannon et al., 2003).

### Quantitative RT-PCR validation

To confirm the accuracy of the high-throughput sequencing results, the qRT-PCR analysis was performed to quantify the transcript levels of randomly selected genes. Total RNA was isolated from tea samples as described before for the samples used in transcriptome sequencing, and cDNA was synthesized using PrimeScript™ RT Reagent Kit (Takara, Dalian, China). The qRT-PCR assay was performed in triplicate on the QuantStudio 3 Real-Time PCR System (Applied Biosystems, Carlsbad, CA, USA) using the TB Green™ Premix Ex Taq™ II Kit (Takara, Dalian, China). All primers used for the qRT-PCR analysis were designed by the automated primer design tool in the Primer Premier (v5.0) software (Premier Biosoft International, Palo Alto, CA, USA) and the DNAMAN (v8.0) software (Lynnon Biosoft, Quebec, Canada). Two commonly used reference genes, *glyceraldehyde-3-phosphate dehydrogenase* (*GAPDH*, TEA003029) and β*-actin* (TEA019484) were used to normalize the relative transcript levels of selected genes in each sample (Wei et al., 2018). ALL primers information was listed in Supplementary Table S1. The relative expression levels of randomly selected genes were calculated with the 2^−ΔΔCT^ method (Livak and Schmittgen, 2001). The above experiments were carried out in accordance with reagent kit instructions and instrument operating manuals.

### Data analyses

The heatmaps of selected DEGs were constructed by using the TBtools (v1.68.0) software (Chen et al., 2020). The GraphPad Prism (v8.0.2) software (GraphPad Software, Inc., San Diego, CA, USA) was used to perform statistical analyses. Group differences were assessed by one-way analysis of variance (ANOVA) followed by Tukey's *post-hoc* test using SPSS (v25.0) software (SPSS Inc., Chicago, USA).

## Results

### Morphological characterization of tea plant trichome

**Figure 1:**
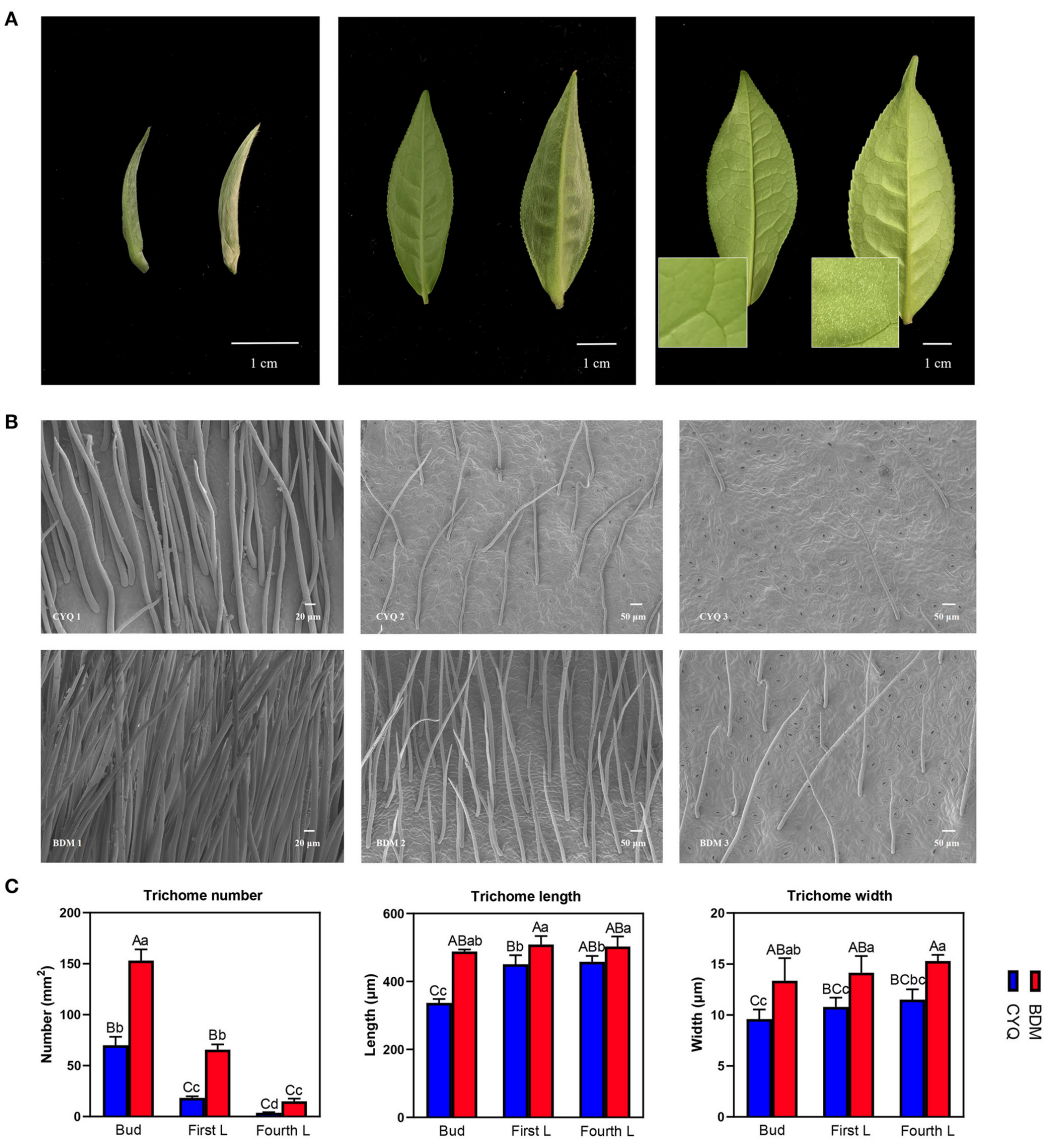
Morphological analyses of trichomes in tea plant cultivars CYQ and BDM. **(A)** Digital photograph of the apical buds, first leaves, and fourth leaves of CYQ and BDM (Left: CYQ; Right: BDM; Scale bar = 1 cm). **(B)** Scanning electron microscopy analysis of trichomes on the apical buds, first leaves, and fourth leaves (left to right) of CYQ (top) and BDM (bottom) (Apical buds: scale bar = 20 μm; first leaves and fourth leaves: scale bar = 50 μm). **(C)** Quantitative analysis of the density, length, and width of trichomes on the apical buds, first leaves, and fourth leaves of CYQ and BDM. The experimental data were herein presented as mean ± standard (SD) deviation for the three or more independent biological replicates. The significant differences among various groups were indicated as uppercase letters (*p* < 0.01) and lowercase letters (*p* < 0.05).

Trichomes are specialized epidermal cells, usually present on the leaves, stems, inflorescence stems, and flowers of most terrestrial plants (Chopra et al., 2019). In this study, BDM cultivar exhibits large, dense, and long trichomes on the apical bud and abaxial surfaces of the young leaf, and these trichomes were even present on the fourth leaf (Figure 1A). In another tea cultivar CYQ, the apical bud and first leaf were unevenly covered with trichomes; however, the fourth leaf was entirely glabrous (Figure 1A). Further, we conducted an SEM assay to characterize the differences in trichome morphological characteristics between two tea cultivars. Trichomes in both tea cultivars were unicellular, unbranched, straight, and soft-structured (Figure 1B), but there were significant differences in the trichome number, length, and width between the two cultivars (Figure 1C). The trichome number (TN) was highest in buds, followed by first and fourth leaves. The trichome length (TL) on buds was significantly lower than on the first and fourth leaves. The trichome width (TW) did not show significant differences among the buds, first leaves, and fourth leaves, although there was an upward trend from the buds to the fourth leaves (Figure 1C).

### Transcriptomic data analysis, DEGs identification and validation

**Figure 2:**
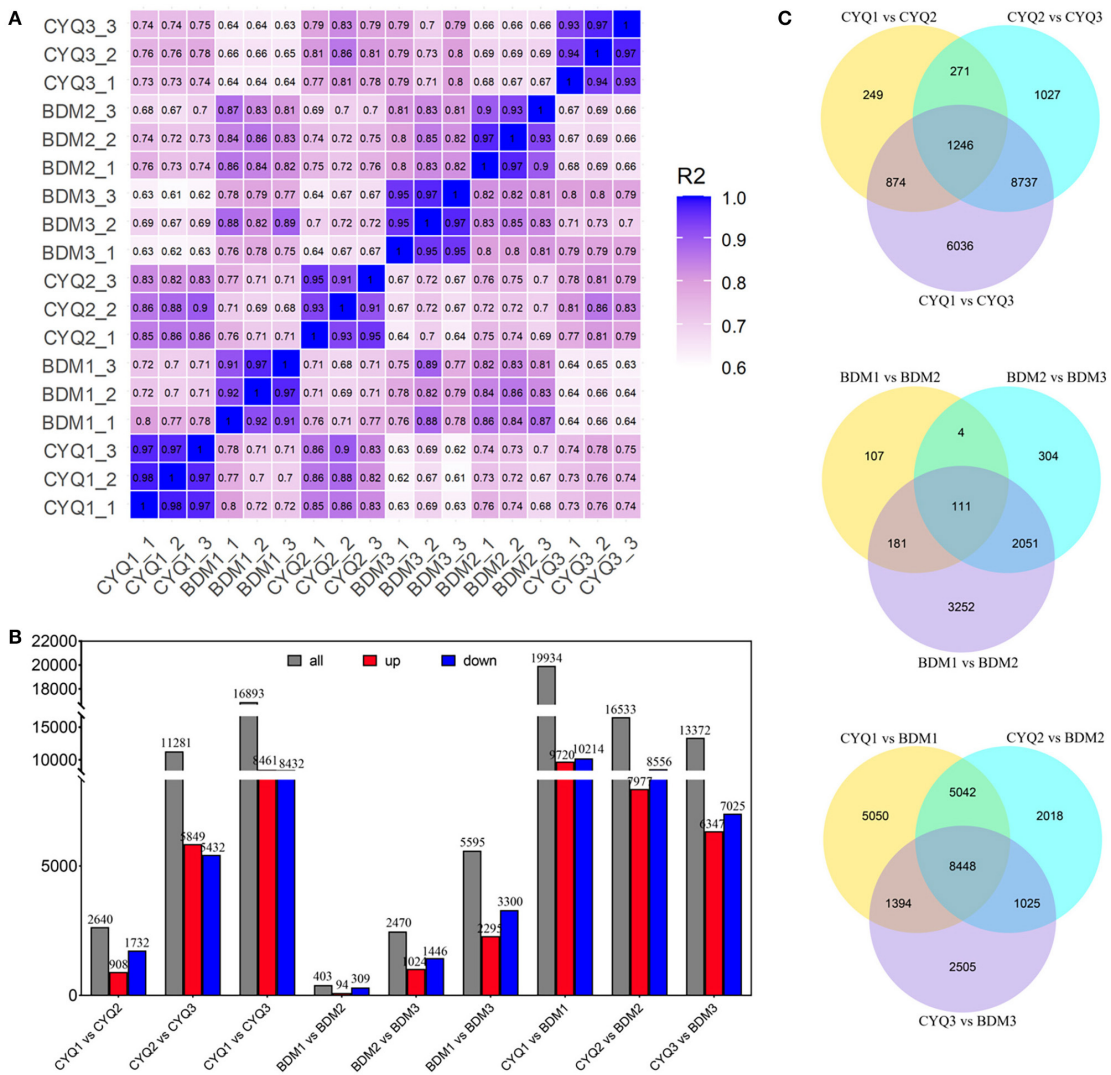
RNA-seq data expression patterns of CYQ and BDM samples. **(A)** Heatmap of squared multiple correlation coefficient of the expression of all transcripts between every two samples. **(B)** The number of up- and down-regulated DEGs in the different compared combinations. **(C)** Venn diagram of co-expressed and uniquely expressed DEGs for the pairwise comparisons.

Using total RNA of CYQ1, CYQ2, CYQ3, BDM1, BDM2, and BDM3 (each sample was repeated three times), we constructed 18 cDNA libraries and sequenced these separately by Illumina sequencing. An average of 9.9 Gb clean bases per library were generated with a mean Q30 level of 93.6% (Supplementary Table S2). The read mapping ratio per sample to the reference genome was 86.22–90.33%, and the average exon, intron, and intergenic regions for each sample were 61.45, 10.32, and 28.24%, respectively (Supplementary Table S3). The coefficient of determination (or squared multiple correlation coefficient, R^2^) values for FPKM between three biological replicates were 0.9 ≤ R^2^ ≤ 0.98, indicating good reproducibility (Figure 2A).

**Figure 3:**
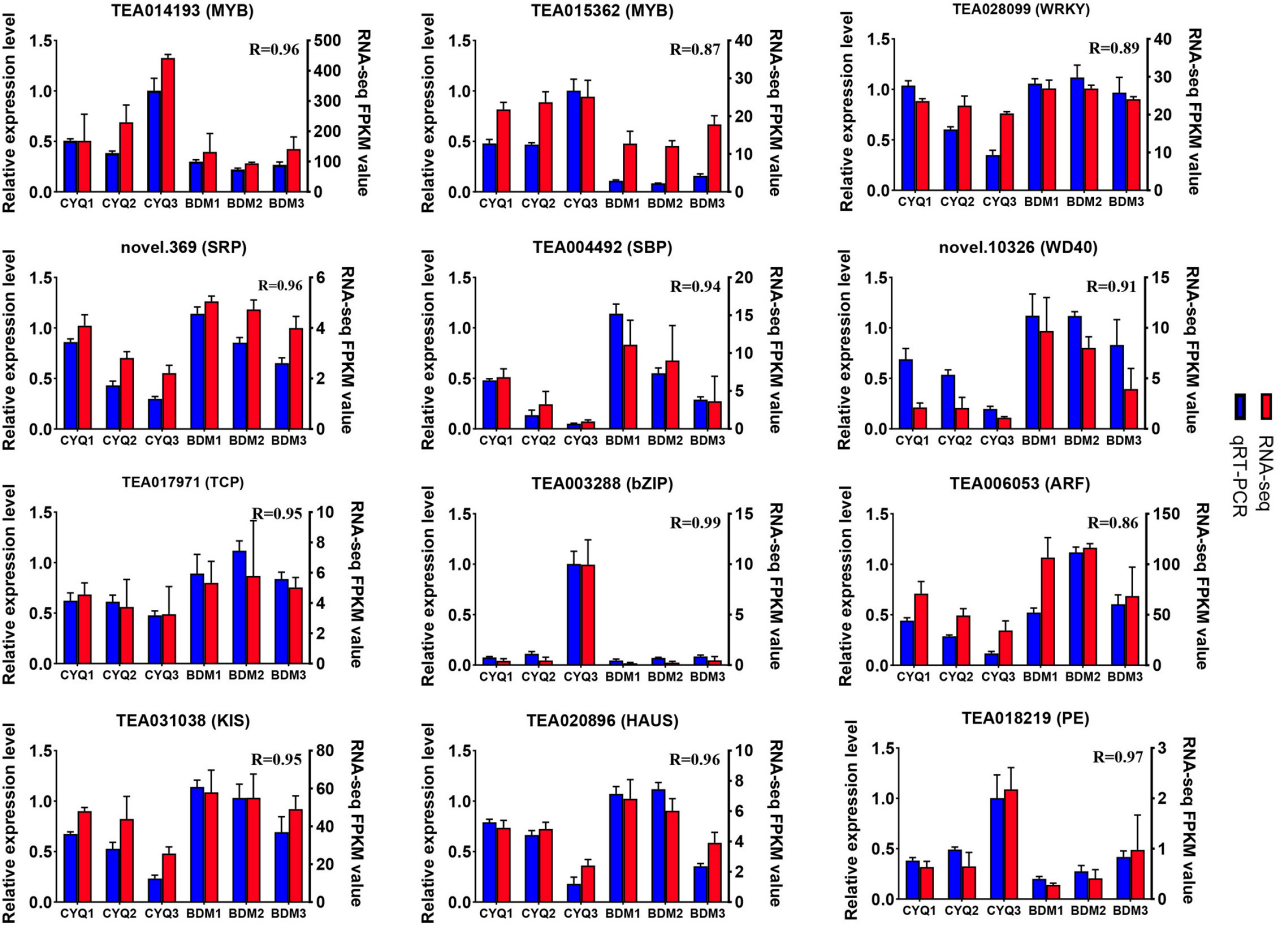
Quantitative RT-PCR validation. Twelve DEGs were selected for qRT-PCR detection. Error bars indicated the standard deviation of the three independent replicates.

We further identified 48,856 unigenes, 31,574 of which were differentially expressed in the nine pairwise comparisons. Among them, there were 2,640 DEGs (908 up-regulated, 1,732 down-regulated) between CYQ1 and CYQ2 comparison, 11,281 DEGs (5,849 up-regulated, 5,432 down-regulated) between CYQ2 and CYQ3 comparison, 16,893 DEGs (8,461 up-regulated, 8,432 down-regulated) between CYQ1 and CYQ3 comparison, 403 DEGs (94 up-regulated, 309 down-regulated**)** between BDM1 and BDM2 comparison, 2,470 DEGs (1,024 up-regulated, 1,446 down-regulated) between BDM2 and BDM3 comparison, 5,595 DEGs (2,295 up-regulated, 3,300 down-regulated) between BDM1 and BDM3 comparison, 19,934 DEGs (9,720 up-regulated, 10,214 down-regulated) between CYQ1 and BDM1 comparison, 16,533 DEGs (7,977 up-regulated, 8,556 down-regulated) between CYQ2 and BDM2 comparison, and 13,372 DEGs (6,347 up-regulated, 7,025 down-regulated) between CYQ3 and BDM3 comparison (Figure 2B). To validate the different expression patterns observed in transcriptomic data, 12 DEGs were randomly selected for qRT-PCR investigation (Figure 3). Results showed that the change expression abundances of selected genes obtained by qRT-PCR were consistent with that of RNA-seq data, with the correlation coefficient (R) values above 0.85. These results confirmed the reliability and accuracy of the transcriptomic data in this study.

### Venn analysis of the DEGs

We performed the Venn diagram analysis to identify the possible genes of tea plant trichome initialization, growth, and development. The Venn diagram represented the number of specific and overlapping DEGs (Figure 2C). A total of 1,246 common DEGs were observed for the three pairwise comparisons of CYQ1 vs. CYQ2, CYQ2 vs. CYQ3, and CYQ1 vs. CYQ3, 111 common DEGs were identified in BDM1 vs. BDM2, BDM2 vs. BDM3, and BDM1 vs. BDM3 pairwise comparisons, and 8,448 common DEGs were identified in CYQ1 vs. BDM1, CYQ2 vs. BDM2, and CYQ3 vs. BDM3 pairwise comparisons (Figure 2C). After filtering the duplicate DEGs from three comparisons, 9,629 DEGs were selected, 4,142 of which were assigned to 1,943 GO terms (Supplementary Table S4). GO enrichment analysis was divided into three major GO categories, namely, biological process (BP), cellular component (CC), and molecular function (MF). Based on a previous study (Doroshkov et al., 2019), we found that trichome formation and development may be related to 103 GO terms, including 71 in BP category, 14 in CC category, and 18 in MF category (Supplementary Table S5). These GO terms were mainly related to TFs, cytoskeleton structure, cell wall structure, cell cycle, hormone regulation, and other trichome related-GO terms (Supplementary Table S5).

### GO enrichment analysis of the DEGs

**Figure 4:**
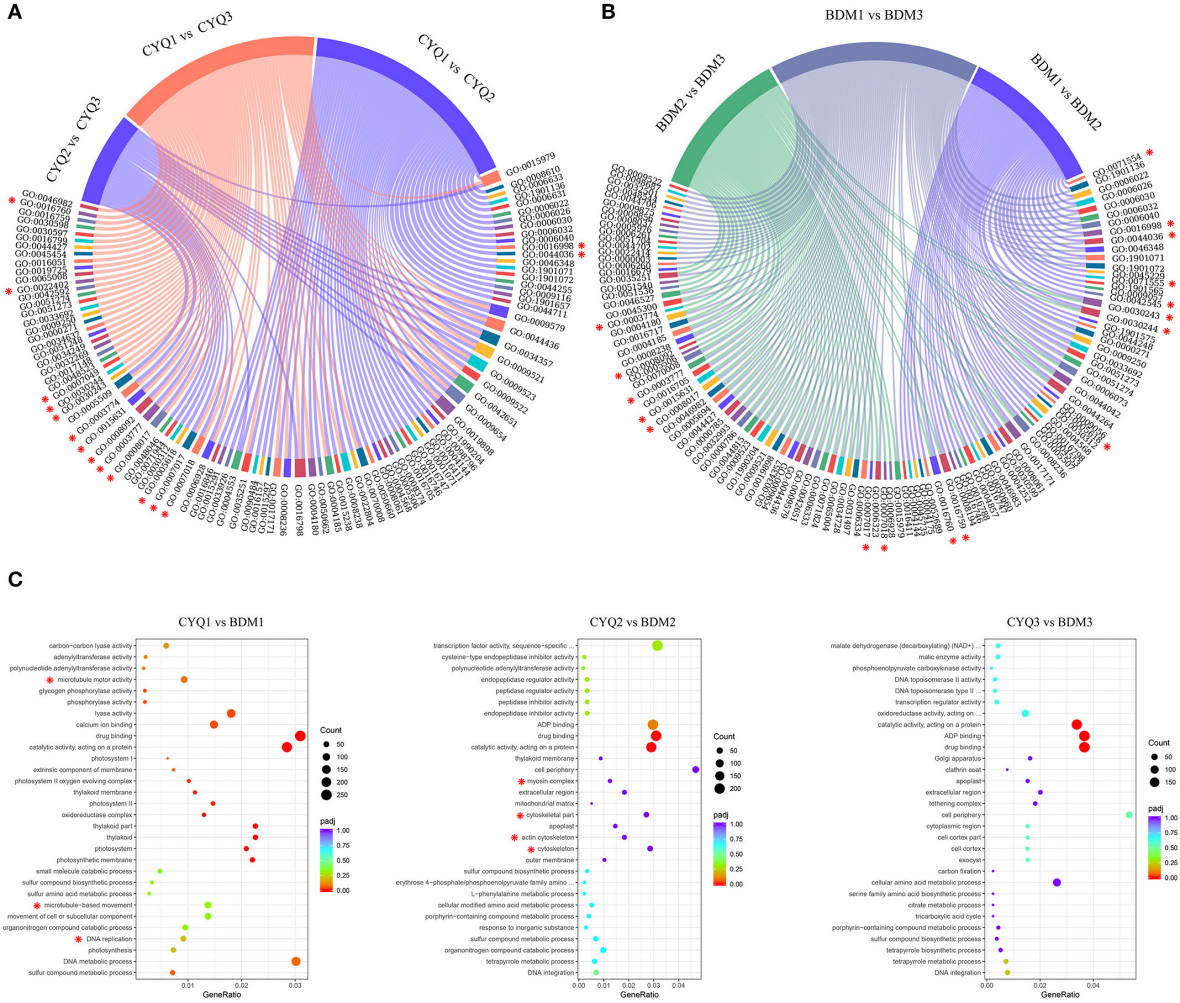
GO enrichment analysis of DEGs. **(A)** Overall enrichment terms of DEGs of CYQ1 vs. CYQ2, CYQ2 vs. CYQ3, and CYQ1 vs. CYQ3 comparisons. **(B)** Overall enrichment terms of DEGs of BDM1 vs. BDM2, BDM2 vs. BDM3, and BDM1 vs. BDM3 comparisons. **(C)** The top 10 significantly enriched GO terms of BP, CC, and MF categories of CYQ1 vs. BDM1, CYQ2 vs. BDM2, and CYQ3 vs. BDM3 comparisons.

We further analyzed the significant enriched GO terms of three main GO categories of each comparison to distinguish the differences in the trichome development between the hairless CYQ and the hairy BDM cultivars. In the CYQ group, the significantly enriched GO categories of the CYQ1 vs. CYQ2, CYQ2 vs. CYQ3, and CYQ1 vs. CYQ3 comparisons were 59, 24, and 54, respectively (Supplementary Table S6A). In the CYQ1 vs. CYQ2 comparison, GO terms of cell wall macromolecule catabolic process (GO:0016998) and cell wall macromolecule metabolic process (GO:0044036) of BP category may participate in the growth and development process of tea plant trichomes (Figure 4A; Supplementary Table S6A). Regarding the CYQ2 vs. CYQ3 comparison, eight GO terms of three main GO categories, including microtubule-based movement (GO:0007018), microtubule-based process (GO:0007017), cell wall (GO:0005618), microtubule binding (GO:0008017), microtubule motor activity (GO:0003777), cytoskeletal protein binding (GO:0008092), tubulin binding (GO:0015631), and motor activity (GO:0003774) related to trichome development (Figure 4A; Supplementary Table S6A). In the CYQ1 vs. CYQ3 comparison, six significantly enriched GO terms of microtubule-based movement (GO:0007018), microtubule-based process (GO:0007017), cellulose metabolic process (GO:0030243), cellulose biosynthetic process (GO:0030244), cell cycle (GO:0007049), and cell cycle process (GO:0022402) in BP category, and seven in MF category, including microtubule binding (GO:0008017), tubulin binding (GO:0015631), microtubule motor activity (GO:0003777), cytoskeletal protein binding (GO:0008092), cellulose synthase activity (GO:0016759), cellulose synthase (UDP-forming) activity (GO:0016760), and motor activity (GO:0003774) related to the trichome development process (Figure 4A; Supplementary Table S6A). In total, 15 GO terms related to the trichome in the development process of the hairless CYQ tea cultivar, including “cell wall,” “cytoskeleton structure,” and “cell cycle”-related GO terms (Figure 4A; Supplementary Table S6A).

In the GO annotation of the hairy BDM cultivar, the significantly enriched GO categories of the BDM1 vs. BDM2, BDM2 vs. BDM3, and BDM1 vs. BDM3 comparisons were 55, 57, and 78, respectively (Supplementary Table S6B). In the BDM1 vs. BDM2 comparison, ten GO subcategories related to trichomes were as follow: cell wall organization or biogenesis (GO:0071554), cell wall macromolecule catabolic process (GO:0016998), cell wall macromolecule metabolic process (GO:0044036), cell wall organization (GO:0071555), cell wall modification (GO:0042545), cellulose metabolic process (GO:0030243), cellulose biosynthetic process (GO:0030244), cell wall (GO:0005618), cellulose synthase activity (GO:0016759), and cellulose synthase (UDP-forming) activity (GO:0016760) (Figure 4B; Supplementary Table S6B). In the BDM2 vs. BDM3 comparison, GO terms of BP and MF categories, including: microtubule-based movement (GO:0007018), microtubule-based process (GO:0007017), cellulose metabolic process (GO:0030243), cellulose biosynthetic process (GO:0030244), microtubule binding (GO:0008017), tubulin binding (GO:0015631), microtubule motor activity (GO:0003777), cytoskeletal protein binding (GO:0008092), motor activity (GO:0003774), cellulose synthase activity (GO:0016759), and cellulose synthase (UDP-forming) activity (GO:0016760) played important roles in trichome development (Figure 4B; Supplementary Table S6B). Furthermore, in the BDM1 vs. BDM3 comparison, nine GO subcategories of cell wall macromolecule catabolic process (GO:0016998), cell wall macromolecule metabolic process (GO:0044036), cellulose metabolic process (GO:0030243), cellulose biosynthetic process (GO:0030244), microtubule binding (GO:0008017), tubulin binding (GO:0015631), cellulose synthase activity (GO:0016759), cellulose synthase (UDP-forming) activity (GO:0016760), and microtubule motor activity (GO:0003777) related to tea plant trichome growth (Figure 4B; Supplementary Table S6B). In total, 17 GO terms related to trichomes in the development process of hairy BDM tea plant cultivar, including “cell wall” and “cytoskeleton structure”-related GO terms (Figure 4B; Supplementary Table S6B). Compared to the hairless CYQ cultivar, more “cellulose” and “cell wall”-related GO terms were found in the hairy BDM cultivar, which may be why the trichomes of the hairy BDM cultivar were more abundant than that of the hairless CYQ cultivar.

We further analyzed the top 10 BP, CC, and MF categories of GO annotation from the development process of the hairless CYQ and the hairy BDM tea cultivars, including the CYQ1 vs. BDM1 comparison, the CYQ2 vs. BDM2 comparison, and the CYQ3 vs. BDM3 comparison. There were three subcategories of DNA replication (GO:0006260), microtubule-based movement (GO:0007018), and microtubule motor activity (GO:0003777) related to trichome from the CYQ1 vs. BDM1 comparison (Figure 4C; Supplementary Table S6C). In the CYQ2 vs. BDM2 comparison, four GO terms related to trichome were as follow: cytoskeleton (GO:0005856), actin cytoskeleton (GO:0015629), cytoskeletal part (GO:0044430), and myosin complex (GO:0016459) (Figure 4C; Supplementary Table S6C). However, no subcategories were related to trichome development in the CYQ3 vs. BDM3 comparison. In short, the genes involved in the GO terms of “DNA replication” and “cytoskeleton” were significantly differentially expressed from apical buds and first leaves stages in the hairless CYQ and the hairy BDM tea cultivars, indicating that these processes may be important for the early development of tea plant trichomes.

### The TFs involved in the regulation of tea plant trichome

**Figure 5:**
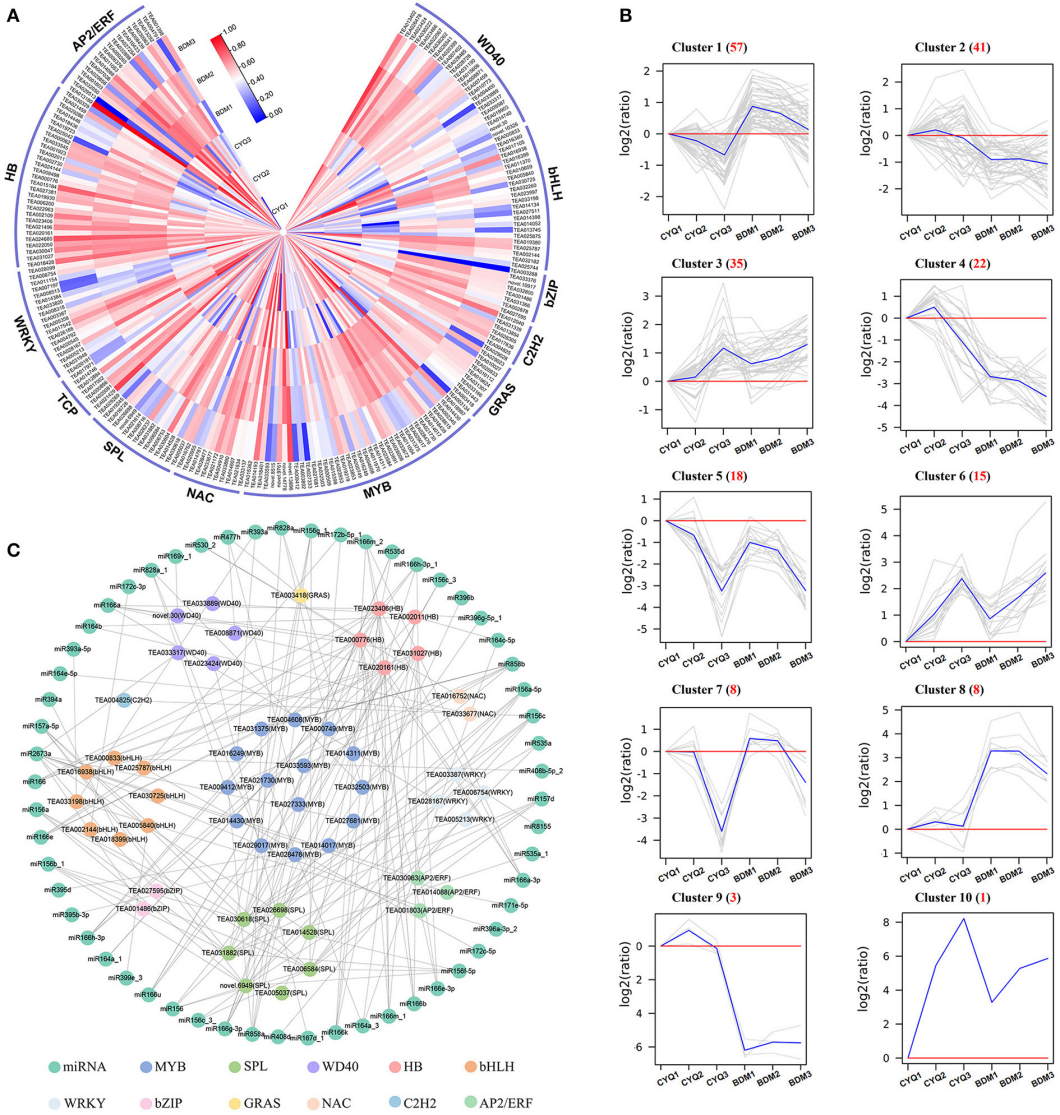
The analyses of differentially expressed TFs. **(A)** The expression patterns of differentially expressed TFs. The expression levels of DEGs were normalized by log_2_ (FPKM). **(B)** The clustering of differentially expressed TFs. Expression ratios were expressed as log_2_ (ratio). **(C)** The TF-miRNA network of all the identified TFs.

Many TFs are involved in regulating the initiation, growth and development of plant trichomes (Wang et al., 2021). To further illuminate the potential function of TFs in tea plant trichomes, we systematically identified DEGs encoding TFs in the present study. A total of 208 TFs from 12 TF families were identified to be differentially expressed, including AP2/ERF, HB, WRKY, TCP, SPL, NAC, MYB, GRAS, C2H2, bZIP, bHLH, and WD40 (Figure 5A). According to the transcriptome profiles, these TFs exhibited mainly ten expression patterns using the H-clust algorithm (Guo et al., 2011), and the ten clusters were numbered in descending order of the gene number assigned to each (Figure 5B). A total of 126 DEGs encoding TFs in clusters 1, 3, 5, 6, 7, and 10 displayed consistently upregulated or downregulated expression trends in the hairy CYQ and the hairless BDM tea cultivars, indicating that they might function as activators or repressors in tea plant development. Among them, we found five differentially expressed TFs may involve in trichome development, including *MYB75* (TEA011004), *NOK* (TEA033672), *ATML1* (TEA006200), *SPL6* (TEA031882), and *SPL12* (TEA006584) (Supplementary Table S7).

In addition, a previous study demonstrated that miRNAs could regulate plant trichome development indirectly by acting on TFs (Wang et al., 2021). We then mapped identified TFs to the miRNAs reported in tea plants (Guo et al., 2017; Jeyaraj et al., 2017a,b). A total of 53 identified TFs and 57 miRNAs were included in the network. Our analysis predicted multiple miRNA interactions with each TF (Figure 5C). The miRNA showing most interactions included members of miR156, miR157, miR164, miR166, miR171, miR172, miR535, miR828, miR858, miR396, and miR2673 families (Supplementary Table S8).

### The DEGs involved in the stress defense

**Figure 6:**
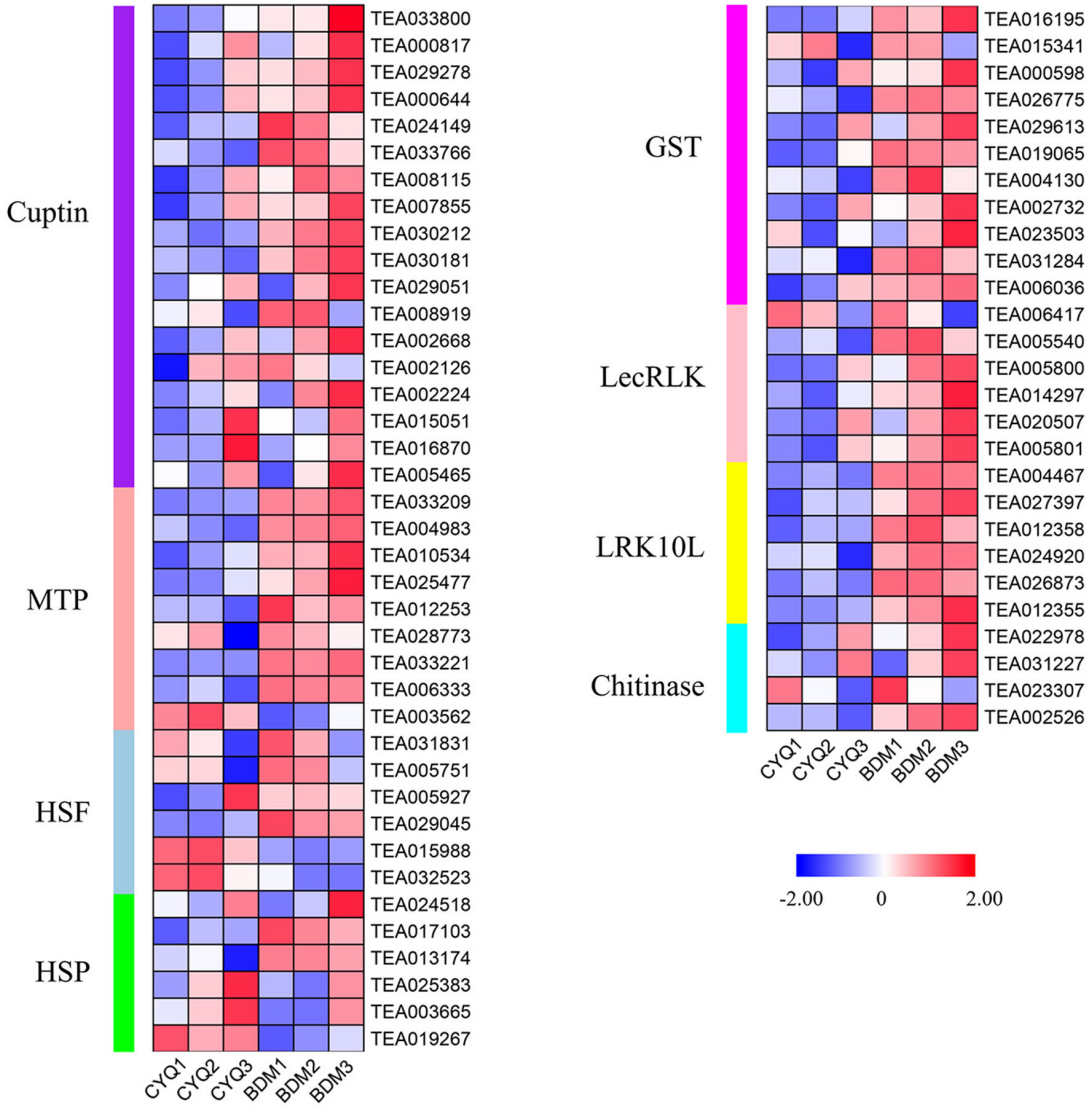
The expression pattern of plant resistance genes (PRGs). DEGs related to PRGs. The expression levels of DEGs were normalized by log_2_ (FPKM).

In addition, 66 DEGs related to plant resistance genes (PRGs) were found in this study, including 18 *cupin superfamily proteins* (*Cupin*), nine *metal tolerance protein* (*MTP*), six *heat shock factor* (*HSF*), six *heat shock protein* (*HSP*), 11 *glutathione S-transferase* (*GST*), six *lectin receptor-like kinases* (*LecRLK*), six *leaf rust 10 disease-resistance locus receptor-like protein kinase* (*LRK10L*) and four *chitinase* (Figure 6). Expression analysis showed that these DEGs were highly expressed in the BDM group than in the CYQ group (Figure 6).

### The DEGs involved in the cell wall pathway

**Figure 7:**
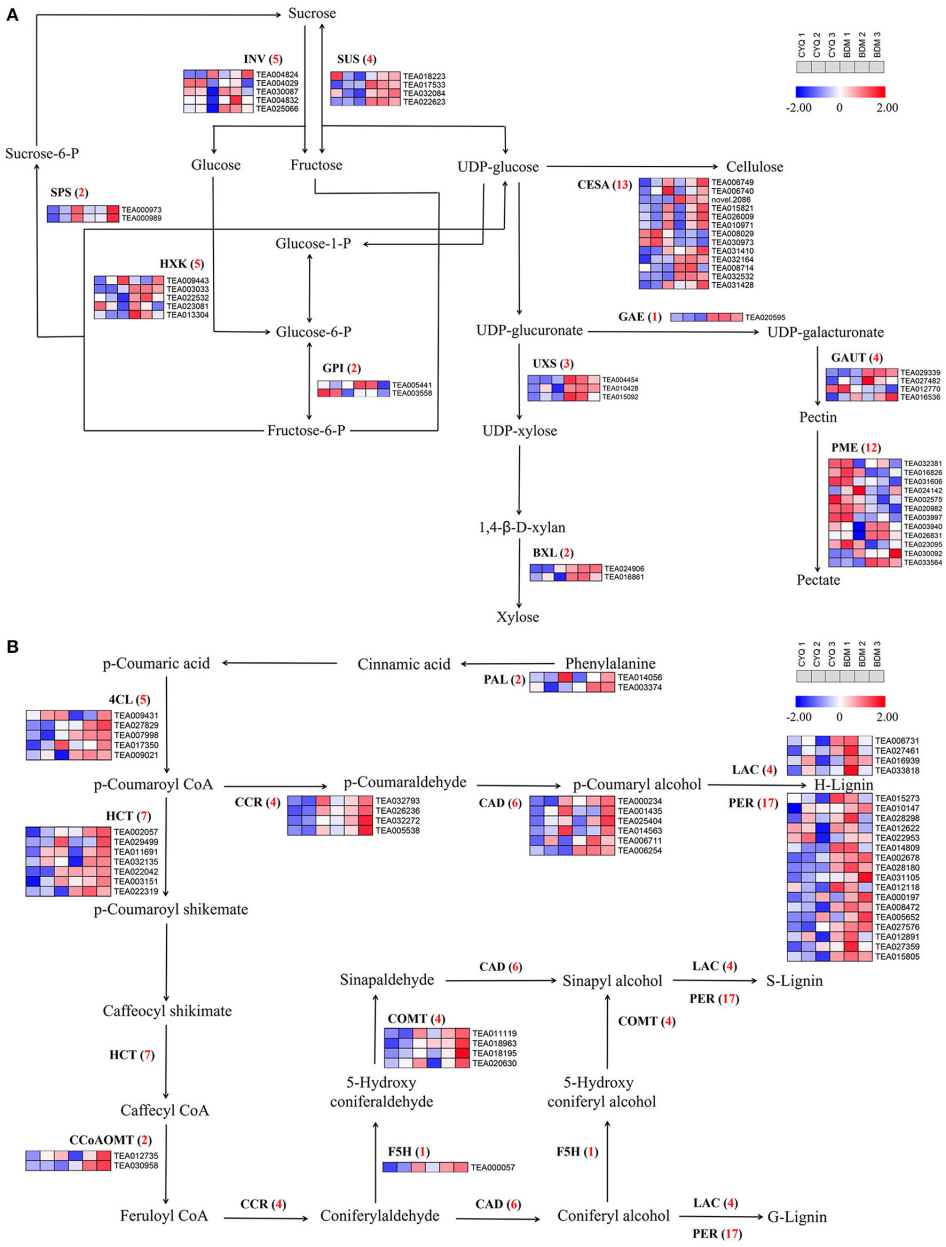
The cellulose, pectin, and lignin biosynthesis pathway and related gene expressions. **(A)** DEGs involved in cellulose and pectin biosynthesis pathway. Numbers in parentheses following each gene name indicated the number of corresponding DEGs. The expression levels of DEGs were normalized by log_2_ (FPKM). **(B)** DEGs involved in lignin biosynthesis pathway. Numbers in parentheses following each gene name indicated the number of corresponding DEGs. The expression levels of DEGs were normalized by log_2_ (FPKM).

**Figure 8:**
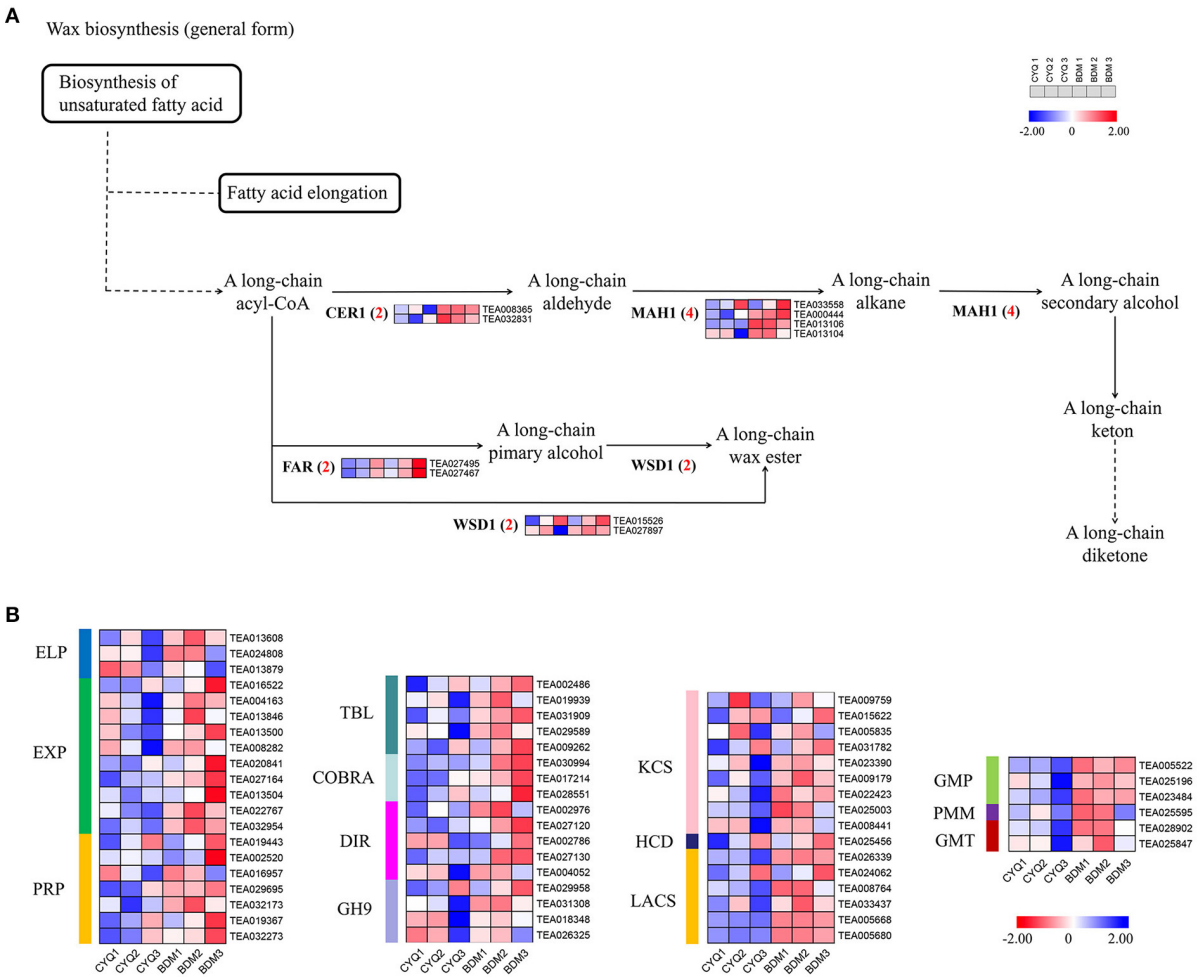
The expression pattern of cuticular wax biosynthesis pathway. **(A)** DEGs involved in cuticular wax biosynthesis pathway. Numbers in parentheses following each gene name indicated the number of corresponding DEGs. The expression levels of DEGs were normalized by log_2_ (FPKM). **(B)** Heatmap of other cell wall material-related genes. The expression levels of DEGs were normalized by log_2_ (FPKM).

In the present study, GO enrichment analysis of DEGs revealed that “cell wall” and “cellulose”-associated terms were significantly overrepresented in the hairy BDM cultivar (Figure 4). Therefore, we further identified the key pathways and genes in the metabolism of trichome cell wall components, including pectin, cellulose, lignin, MCPs, and cuticular wax from the hairless CYQ and the hairy BDM cultivars. In total, we identified 51 DEGs involved in the pectin and cellulose metabolism pathway, including five *invertase* (*INV*), four *sucrose synthase* (*SUS*), two *sucrose phosphate synthase* (*SPS*), five *hexokinase* (*HXK*), two *glucose-6-phosphate isomerase* (*GPI*), 13 *CESA*, three *UDP-xylose synthase* (*UXS*), two β*-D-xylosidase* (*BXL*), one *UDP-glucuronate 4-epimerase* (*GAE*), four *galacturonosyl-transferase* (*GAUT*), and ten *pectin methylesterase* (*PME*) (Figure 7A). In addition, we identified 52 DEGs involved in the lignin biosynthesis pathway, including two *PAL*, five *4CL*, four *CCR*, six *CAD*, seven *HCT*, two *CCoAOMT*, one *F5H*, four *COMT*, four *LAC*, and 17 *PER* (Figure 7B). Meanwhile, dirigent (DIR), glycoside hydrolase 9 (GH9), COBRA, TBL also play important roles in cellulose and lignin biosynthesis in plant trichomes (Bischoff et al., 2010; Xie et al., 2013; Niu et al., 2015; Liu et al., 2021). In this work, five *TBL*, three *COBRA*, five *DIR*, and four *GH9* were differentially expressed (Figure 8B). Moreover, we found differential expression of three *GDP-mannose pyrophosphorylase* (*GMP*), one *phosphomannomutase* (*PMM*), and two *GMT* (*GDP-mannose transporter*), which are known to be involved in MCPs biosynthesis (Figure 8B). Moreover, KCS, HCD, and LACS have been implicated in trichome wax formation (Hegebarth et al., 2016). The present study identified nine *KCS*, one *HCD*, and six *LACS* as differentially expressed. Also, ten DEGs were involved in the wax biosynthesis pathway, including two *CER1*, four *MAH1*, two *FAR*, and two *WSD1* (Figure 8A). Other cell wall-related genes, like *extensin-like protein* (*ELP*), *expansin* (*EXP*), and *proline-rich protein* (*PRP*), are also essential for cell wall formation (Li et al., 2020b). In this study, three *ELP*, ten *EXP*, and seven *PRP* showed significantly differential expression trends in our work (Figure 8B). Notably, expression analysis showed that the above-identified DEGs were expressed at higher levels in the BDM group than in the CYQ group, which may contribute to the accumulation of cell wall materials in tea plant trichomes (Figures 7, 8).

### The DEGs involved in the cell cycle pathway

**Figure 9:**
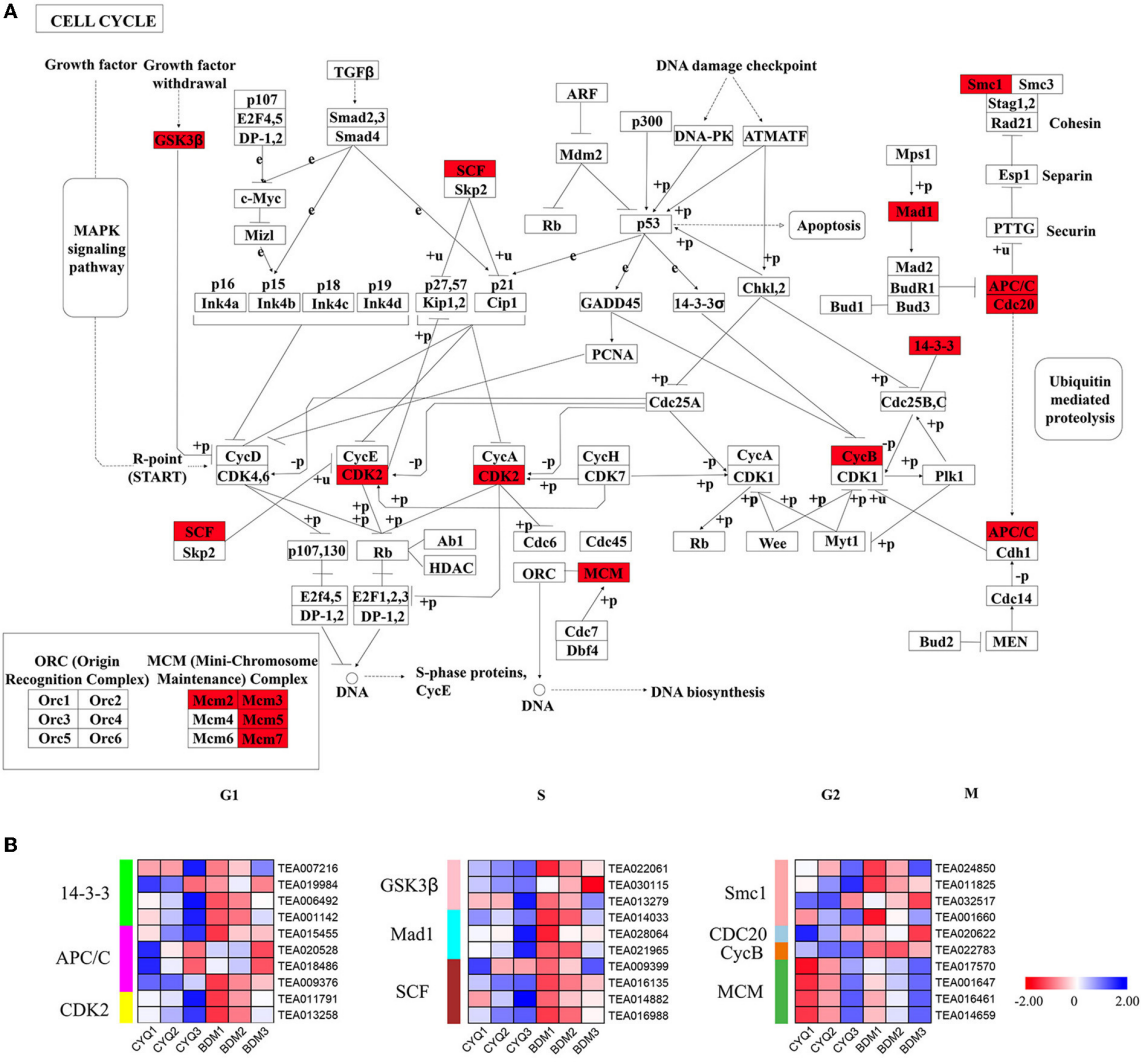
The cell cycle pathway and related gene expressions. **(A)** Cell cycle map of KEGG. Red color indicated DEGs in the cell cycle pathway. **(B)** Expression analysis for DEG associated with the cell cycle. The expression levels of DEGs were normalized by log_2_ (FPKM).

The trichome development is closely related to the cell cycle pathway (Yang and Ye, 2013). In this study, we identified 29 DEGs in the cell cycle pathway, including four *14-3-3*, four *anaphase-promoting complex/cyclosome* (*APC/C*), two *cyclin-dependent kinase 2* (*CDK2*), three *glycogen synthase kinase 3 beta* (*GSK3*β), three MAX dimerization protein 1 (*Mad1*), four *Skp1-Cullin1-Fbox complex* (*SCF*), four *structural maintenance of chromosome 1* (*Smc1*), one *cell division cycle 20* (*CDC20*), one *CycB*, and four *mini chromosome maintenance* (*MCM*) (Figure 9). Except for DEGs encoding MCM, other DEGs were expressed at higher levels in the hairy BDM group than in the hairless CYQ group (Figure 9).

### The DEGs involved in the cytoskeleton structure

**Figure 10:**
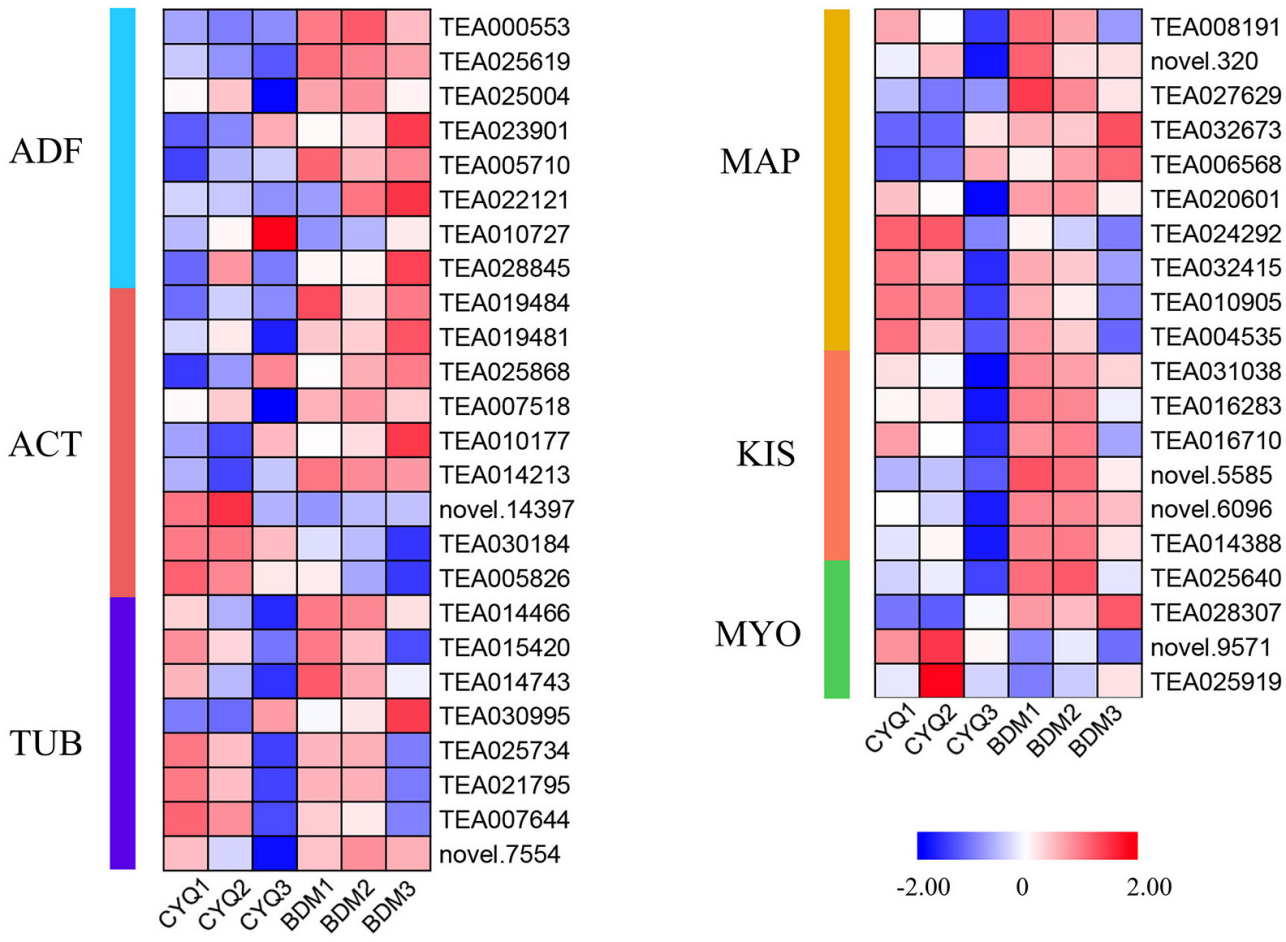
The expression pattern of cytoskeleton biosynthesis related genes. DEGs related to cytoskeleton biosynthesis. The expression levels of DEGs were normalized by log_2_ (FPKM).

In this study, GO enrichment analysis of DEGs revealed that “cytoskeleton”-associated terms were significantly overrepresented (Figure 4). A total of 45 DEGs related to cytoskeleton structure, including nine *ACT*, eight *TUB*, eight *ADF*, ten *MAP*, six *KIS*, and four *MYO* were identified (Figure 10). We discovered that more DEGs had higher expression in the bud than in the first and fourth leaves. Further, the overall expression levels of the hairy BDM group were higher than that of the hairless CYQ group.

### Co-expressed genes related to trichome growth traits

**Figure 11:**
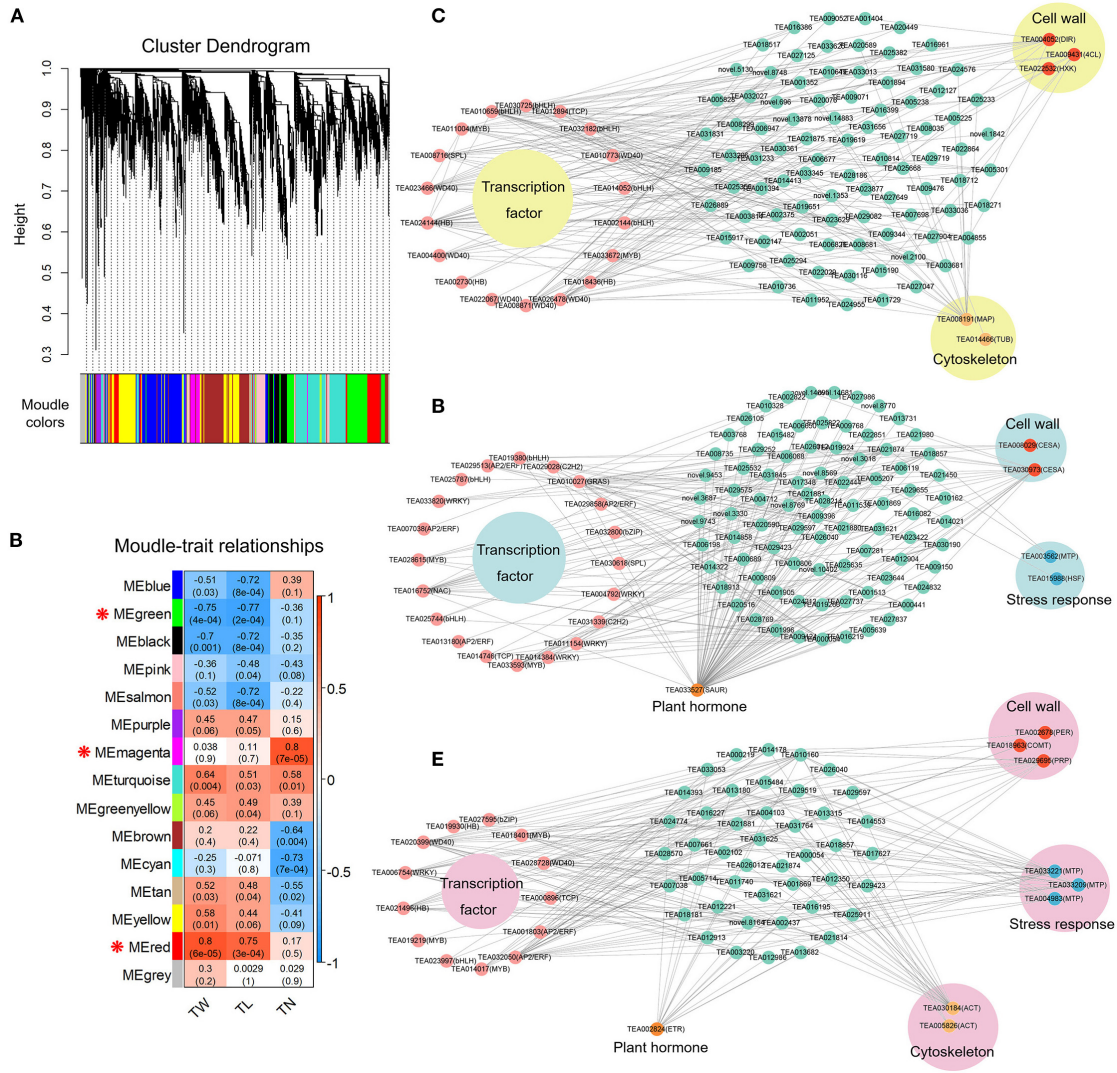
Co-expression network analysis of tea plant trichome development. **(A)** Hierarchical clustering tree of the modules based on WGCNA analysis. **(B)** Relationships between modules and key traits in tested samples. **(C)** Co-expression network diagram of candidate genes in ME magenta module. **(D)** Co-expression network diagram of candidate genes in ME green module. **(E)** Co-expression network diagram of candidate genes in ME red module.

The WGCNA captured gene sets related to tea plant trichome growth traits (TN, TL, and TW) based on 13,306 DEGs and phenotypic data. Fifteen distinct co-expression modules (labeled by 15 different colors) of WGCNA were generated herein that contained highly interconnected gene clusters with high correlation coefficients among genes in the same cluster (Figure 11A). Each module contained positively and negatively correlated genes, and the expression pattern changed between different morphological traits (Figure 11B). The ME magenta module (333) was closely connected with TN (*r* = 0.8, *P-*value = 7e-05). In addition, the ME green module (1130) presented a dramatically high negative correlation with TW (*r* = −0.75, *P-*value = 4e-04) and TL (*r* = −0.77, *P-*value = 2e-04), and the ME red module (831) showed a significant positive correlation with TW (*r* = 0.8, *P-*value = 6e-05) and TL (*r* = 0.75, *P-*value = 3e-04). These results indicated that the genes belonging to the three modules were most likely involved in regulating trichome formation and development in tea plants.

Further, the top 200 unigene pairs in the ME magenta, ME green, and ME red modules were used to construct the co-expression network, respectively, and the TFs and trichome-related genes were selected as key hub genes. A total of 25 key hub genes were identified in the ME magenta module, including seven *WD40* TF genes, five *bHLH* TF genes, three *HB* TF genes, two *MYB* TF genes, one *bZIP* TF gene, one *SPL* TF gene, and one *TCP* TF gene (Figure 11C). Besides, three key genes (*DIR, 4CL*, and *HXK*) in the cell wall biosynthesis pathway, and two key genes (*MAP* and *TUB*) in the cytoskeleton were also identified (Figure 11C). For the ME green module, 25 genes were selected as key hub genes. Among them, 20 were TFs genes, including four *AP2/ERF*, four *WRKY*, three *bHLH*, two *MYB*, two *C2H2*, one *bZIP*, one *SPL*, one *NAC*, one *GRAS*, and one *TCP* (Figure 11D). In addition, two *CESA* genes in the cell wall biosynthesis pathway, one *MTP* gene and one *HSF* gene in the stress response, and one *SAUR* gene in the plant hormone were identified (Figure 11D). For the ME red module, 22 key hub genes were selected. Among them, 13 were TFs genes, including three *MYB*, two *AP2/ERF*, two *HB*, two *WD40*, one *bHLH*, one *bZIP*, one *WRKY*, and one *TCP* (Figure 11E). Furthermore, one *PER* gene, one *COMT* gene, and one *PRP* gene in the cell wall biosynthesis pathway, three *MTP* genes in the stress response, two *ACT* genes in the cytoskeleton, and one *ETR* gene in the plant hormone were identified (Figure 11E). These findings suggest that the molecular regulation mechanism of tea plant trichome formation is complex and involves many different TF families and other functional genes related to the cell wall, cytoskeleton structure, stress response, and plant hormone.

## Discussion

Tea is processed from postharvest tender leaves from tea plants, and the tea leaves containing more trichomes are considered to produce higher quality tea. Thus, the development of hairy tea cultivars is thus an important goal of breeding programs (Li et al., 2020a). However, a lack of knowledge of the molecular regulatory mechanism underlying the tea trichome morphogenesis process has hindered the exploitation of genetic engineering technologies from accelerating tea genetic improvement. In this study, we performed phenotypic characterization and transcriptome analysis on the hairless CYQ and the hairy BDM tea genotypes to understand trichome development at the morphological and molecular levels.

### Trichome is a crucial characteristic of tea germplasm

Trichomes are the specialized epidermal cells, usually present on the buds, leaves, and stems of tea plants (Li et al., 2020a). Tea plants face a wide range of biotic and abiotic stresses during growth and development. Trichomes, functioning as the first line for plant adaption to environmental stresses, protect tea plants from insect herbivores and pathogen (fungi and bacteria) attacks, reflect UV to avoid physical damages, reduce transpiration, and protect tea leaf from chilling and freezing (Li et al., 2020b, 2022). Besides protecting tea plants as a physical barrier, tea plant trichomes can also secrete various secondary metabolites, including flavonoids, polyphenols, amino acids, purine alkaloids, and volatiles (Cao et al., 2020; Li et al., 2020a,b). Trichomes are unevenly present on the apical buds and abaxial surfaces of tender leaves in most modern tea cultivars of both *Camellia assamica* and *C. sinensis* types (Li et al., 2022). In contrast, most other Thea section plants, including *Camellia taliensis, Camellia angustifolia*, and *Camellia tachangensis* have obvious glabrous leaves or have short and rare trichomes (Li et al., 2022). Thus, long and densely spaced trichomes on the young leaves are important domestication traits in tea germplasm. Trichomes are an extension of the epidermal cells in many plant species, with various structures variations, including unicellular or multicellular, spiral, hooked, or straight, hard or soft, and branched or unbranched (Wang et al., 2021). In the present study, the morphological analysis revealed that trichomes on both the hairless CYQ and the hairy BDM tea cultivars were unicellular, unbranched, straight, and soft types (Figures 1A,B), which was consistent with previous observations in different tea plant cultivars (Yue et al., 2018; Li et al., 2020a,b, 2022). Normally, tea plant trichomes are long (0.4–1 mm) and highly dense, with an average value of 30/mm^2^ (Li et al., 2020b). In our study, the trichome density, length, and width were significantly higher in the hairy BDM than in the hairless CYQ (Figure 1C). In general, the distribution of tea plant trichomes is mainly present on newly budded leaves and gradually decreases as the leaves develop (Sun et al., 2020). However, in the hairy tea cultivar, BDM, trichomes were even present on the fourth leaf (Figures 1A,B). Therefore, BDM may be an important model for studying the mechanisms of trichome formation and development in tea plant species.

### TFs and defensive genes were extensively involved in tea plant trichome development

Tea plant trichome initiation and development are regulated by various genes. To explore the molecular mechanisms involved in the tea plant trichomes development process, transcriptome sequencing of buds, first leaves, and fourth leaves from the hairless CYQ and the hairy BDM tea plant cultivars was carried out. It is known that numerous TFs are involved in regulating the development of plant trichomes (Doroshkov et al., 2019; Wang et al., 2021; Zhang et al., 2021). In the present study, we identified five DEGs encoding TFs that potentially participated in tea plant trichome development based on homology search and expression analysis, including one *MYB75* (TEA011004), one *NOK* (TEA033672), one *ATML1* (TEA006200), one *SPL6* (TEA031882), and one *SPL12* (TEA006584) (Figure 5A; Supplementary Table S7). Of these genes, *MYB75* is a homolog of members of the MBW transcriptional complex that is highly expressed in developing trichomes. MYB75 plays a pivotal role in regulating the initiation and development of trichome by releasing an MBW complex and activating downstream factors (Qi et al., 2011). In our work, the expression level of *MYB75* was significantly increased in the hairy BDM group compared to the hairless CYQ group (Figure 5A; Supplementary Table S7). Moreover, our WGCNA result revealed that *MYB75* is a hub gene associated with trichome density in the ME magenta module (Figure 11C). Therefore, *MYB75* may be essential for initiating and developing tea plant trichomes. *NOK* is encoded by *MYB106*, which belongs to the *MIXTA* subfamily. *MIXTA*, a key regulator gene of petal conical cell form in snapdragon, could trigger trichome formation when ectopically expressed in tobacco (Glover et al., 1998). The tomato *SlMIXTA-like MYB* gene, *SlMX1* is reported to induce leaf and stem trichomes formation, overexpression of *SlMX1* results in a much higher density of trichomes than that on wild-type plants (Ewas et al., 2016). Consistent with these findings, we found that the expression level of *NOK* in the hairy BDM group was higher than that of the hairless CYQ group during the trichome development process (Figure 5A; Supplementary Table S7). Meanwhile, WGCNA results revealed that the expression of *NOK* was highly positively correlated with the development of tea plant trichome number in the ME magenta module (Figure 11C), suggesting that *CsNOK* may positively regulate trichome formation in tea plants. In addition, *ATML1*, belongs to the *HB* subfamily gene. *ATML1* plays a vital role in promoting shoot epidermis differentiation through the regulation of the L1 box-containing genes (Nakamura et al., 2006). A previous study reported that mutations in *ATML1* and its closest homolog *PDF2* lead to the formation of leaves that lack epidermis (Abe et al., 2003). In *Arabidopsis*, overexpression of *ATML1* could activate the expression of epidermal-related genes and induce the formation of trichomes (Takada et al., 2013). In the present study, the expression trend of *ATML1* was consistent with the gradual loss of trichomes in tea plant leaves (Figure 5A; Supplementary Table S7). Moreover, SPL TF is an important negative regulator of trichome development in the floral organ and inflorescence stem (Yu et al., 2010). In our work, many DEGs encoding SPL displayed higher expression patterns in the hairless CYQ than in the hairy BDM, *SPL6* and *SPL12* were significantly up-regulated in the hairless CYQ (Figure 5A; Supplementary Table S7). MiRNAs are a class of small, endogenous, non-coding RNAs of 20-24 nucleotides in length that negatively regulate gene expression (Song et al., 2019). In the plant development process, miRNAs regulate several phenomena, including trichome differentiation and morphogenesis (Fambrini and Pugliesi, 2019). It was proposed that miR156 is a typical graft-transmissible miRNA that modulates trichome density by regulating the expression of its target (Bhogale et al., 2014). In *Arabidopsis*, the SPL TF family contains 17 members, ten of which are targeted by miR156 family members (Rhoades et al., 2002). In alfalfa, overexpressing miR156 displayed increased trichome density by decreasing the expressions of *SPL6, SPL12*, and *SPL13* (Aung et al., 2015). We found that miR156 interacts with *SPL6* and *SPL12* in tea plants, suggesting its importance in trichome development. In addition, other miRNAs, such as miR157 (He et al., 2018), miR171 (Xue et al., 2014), miR319 (Fan et al., 2020), miR828 (Guan et al., 2014), and miR858 (Guan et al., 2014) are known to be involved in trichome development. Notably, these miRNAs were also identified in this study (Figure 5C; Supplementary Table S8), but deciphering the potential roles of miRNAs in tea plant trichome development warrants further investigation.

Simultaneously, plant trichomes frequently function as the first physical barrier of defense against biotic and abiotic stresses through space hindrance. Previous studies found that the expression levels of plant resistance genes (PRGs), such as *Cuptin, MTP, HSF, HSP, GST, LecRLK, LRK10L*, and *Chitinase* were highly upregulated in tea plant trichomes (Cao et al., 2020; Li et al., 2020a, 2022). In this work, we found that most DEGs encoding these PRGs were extensively expressed in the hairy BDM group compared with the hairless CYQ group (Figure 6). Among these, the expressions of three unigenes (TEA033221, TEA033209, and TEA004983) encoding MTP positively correlated with tea plant trichome length and width (Figure 11E). The present data suggested that tea plant trichome-related PRGs may play unrecognized roles in plant defense. These results would align with the generally accepted functions of plant trichomes as protective structures that shield plant organs from various environmental stresses.

### Cell wall materials were associated with tea plant trichome development

With the advantage of the development of high-throughput sequencing technology, we can easily generate a broad view of metabolisms involved in the development of tea plant trichomes. DEGs in our sequencing were significantly enriched in the “cell wall” and “cellulose”-associated GO terms (Figures 4A,B). Cell walls are important for trichomes in supporting morphological variations and defensive functions. Generally, plant trichomes contain various cell wall materials, such as pectin, cellulose, lignin, MCPs, and cuticular wax (Marks et al., 2008). In previous studies, researchers have shown that the genes involved in cell wall biosynthesis, function, and structure were expressed at high levels in trichomes (Jakoby et al., 2008; Marks et al., 2009). In our study, three key enzymes associated with pectin metabolism were identified to differ in transcript abundance between the hairless CYQ and the hairy BDM. Among them, one unigene encoding GAE, which catalyzes the conversion of UDP-glucuronate to UDP-galacturonate, and three unigenes encoding GAUT, which participate in pectic polysaccharide homogalacturonan (Mohnen, 2008), were significantly upregulated in the hairy BDM group than the hairless CYQ group (Figure 7A). In contrast, most DEGs encoding PME, which are important for pectin degradation (Mohnen, 2008), were repressed in the hairy BDM group (Figure 7A). Taken together, the increase in the expression levels of the genes involved in pectin biosynthesis, and the decline in the expression levels of the genes involved in pectin degradation, indicated that pectin content may have increased in tea plant trichomes. In addition to pectin, cellulose is also an important constituent required to establish a thick trichome cell wall (Liang et al., 1993). CESA is a key enzyme for cellulose synthesis (Somerville, 2006) and is critical for expansion and secondary wall thickening in plant shoot trichomes (Betancur et al., 2010). Besides the CESA, several other proteins, such as COBRA, TBL, and GH9 are believed to participate in cellulose synthesis and deposition, and impact trichomes development (Bischoff et al., 2010; Xie et al., 2013; Niu et al., 2015). In the present study, most DEGs encoding CESA (Figure 7A), TBL, COBRA, and GH9 (Figure 8B) were repressed in the CYQ group, most likely, due to its hairless phenotype. These results were consistent with a previous study focusing on tea plant trichomes (Yue et al., 2018).

Plant trichomes usually contain lignin to support their morphological variations and functions (Marks et al., 2008). Lignin biosynthesis is controlled by a set of enzymes, and PAL, C4H, and 4CL regulated the first three steps (Weng and Chapple, 2010). These three enzymes are involved in the processing of lignin and are shared by pathways involved in forming other key secondary metabolites, such as flavonoid, anthocyanin, and coumarin (Ali and McNear, 2014). Overexpression of *GbMYBR1*, a trichomes repressor in *Arabidopsis*, led to reduced trichome density, reduced expressions of lignin-related genes such as *PAL* and *4CL*, and decreased lignin content (Su et al., 2020). In our study, *PAL* and *4CL* were extensively expressed in the hairy BDM compared with the hairless CYQ (Figure 7B), which is consistent with a previous study on tea plant trichomes (Li et al., 2020b). Further, our WGCNA revealed that a unigene (TEA009431) encoding 4CL showed a positive expression trend with trichome density in the ME magenta module (Figure 11C). These observations suggest that *PAL* and *4CL* may control lignin synthesis during trichome development in tea plants. Nevertheless, the *C4H* transcript level did not correlate with lignin accumulation, possibly due to the associations between the phenylpropanoid pathway and flavonoid or anthocyanin and coumarin metabolic pathways. The genes encoding HCT, F5H, CCoAOMT, and COMT are important to lignin synthesis and are involved in the synthesis of sinapyl alcohol (S-unit lignin) and coniferyl alcohol (G-unit lignin). A previous study observed that inhibition of *HCT* gene expression significantly reduced lignin content and changed S-unit lignin content, G-unit lignin content, and S/G ratio in poplar (Zhou et al., 2020a). Also, in *Rosa roxburghii* fruit spines (also called fruit trichomes), the transcriptional levels of *HCT* were significantly positively correlated with the lignin and monomer contents (Lu et al., 2020). Meanwhile, interference in the transcriptions of *F5H, CCoAOMT*, and *COMT* were also found to alter the lignin content (Liu et al., 2018). It has been shown earlier that *HCT, F5H, CCoAOMT*, and *COMT* were expressed at higher levels in tea plant trichomes than in trichome-removed leaves (Li et al., 2020b). In the present study, most *HCT, F5H, CCoAOMT*, and *COMT* were upregulated in the hairy BDM group compared to the hairless CYQ group (Figure 7B). Further, one *COMT* (TEA018963) was a hub gene correlated with tea plant trichome length and width in the ME red module (Figure 11E). These observations indicated that the *HCT, F5H, CCoAOMT*, and *COMT* genes may promote the accumulation of lignin in the hairy tea cultivar. CCR and CAD are the two important enzymes of the monolignol biosynthesis process (Zhao and Dixon, 2011). CCR catalyzes the first committed step of lignin specific branch to produce lignin monomers, while CAD acts on the last step in the formation of monolignols (Zhao and Dixon, 2011). In olive trichomes, the transcriptional levels of the *CCD* and *CCR* were highly induced (Koudounas et al., 2015). The simultaneous suppression of *CCR* and *CAD* gene not only significantly reduced lignin content but also severely affected plant development in *Arabidopsis* (Thevenin et al., 2011). In our study, most DEGs encoding CCD and CCR were highly expressed in the hairy BDM group (Figure 7B). PER and LAC enzymes are also essential for lignin polymerization (Zhao and Dixon, 2011). Several genes encoding PER and LAC have been reported to affect lignin content in *Arabidopsis* (Shigeto et al., 2013, 2014; Zhao et al., 2013). In cotton, *GhLAC1* showed a high expression level in developing fiber cells (Hu et al., 2020). Also, most DEGs encoding PER in the tea plant were significantly upregulated in trichomes (Cao et al., 2020). In our work, the expression levels of most DEGs encoding PER and LAC were upregulated in the hairy BDM group compared to the hairless CYQ group (Figure 7B). Among them, a DEG (TEA002678) encoding PER positively correlated with tea plant trichome length and width of the ME red module in WGCNA results (Figure 11E), indicating that *PER* and *LAC* may play potential roles in the accumulation of lignin content in trichomes. In addition, *DIR* plays an important role in cotton lignin accumulation and impacts cotton fiber development (Liu et al., 2021). We identified five DEGs encoding DIR in this study; their expression levels increased in the hairy BDM group compared to the hairless CYQ group (Figure 8B). Meanwhile, the WGCNA results indicated that a unigene (TEA004052) encoding DIR was the hub gene in the ME magenta module (Figure 11C). Taken together, these results strengthen the possibility that the lignin metabolism pathway is active in tea plant trichomes.

Plant trichomes also contain a certain amount of MCPs and are covered with cuticular wax (Marks et al., 2008). In our analysis, several DEGs encoding PMM, GMP, and GMT that are known to be involved in MCPs biosynthesis were differentially expressed in the hairless CYQ and the hairy BDM (Figure 8B). Among them, PMM catalyzes the conversion of mannose-6-phosphate (M6P) to mannose-1-phosphate (M1P) (Zhang et al., 2018). PMM is a critical regulator of the MCPs metabolic pathway. The ectopic expression of *DoPMM* in *Arabidopsis* remarkably triggered the MCPs accumulation (He et al., 2017b). GMP serves as a donor for the biosynthesis of MCPs and plays a vital role in catalyzing the conversion of M1P to GDP-mannose (Reyes and Orellana, 2008). In *Arabidopsis*, plants overexpressing *DoGMP1* produce much higher mannose content than wild-type plants (He et al., 2017a). In this work, we found that the expression levels of *PMM* and *GMP* were significantly higher in the hairy BDM group (Figure 8B), which may contribute to the high accumulation of MCPs in the hairy BDM tea cultivar. In addition, GMT is indispensable for the biosynthesis of MCPs, which delivers GDP-mannose from the cytosol to the Golgi lumen for glycosylation reactions (Orellana et al., 2016). GONST1, which belongs to the GMT family, is a specific GDP-mannose transporter (Baldwin et al., 2001). In *Dendrobium officinale*, DoGMT1, DoGMT2, and DoGMT3 were showed to transport GDP-mannose, and the transcript abundance of *DoGMT1, DoGMT2*, and *DoGMT3* was strongly correlated with an increase in MCPs content (Yu et al., 2018). In the present study, the DEGs encoding GMT were expressed at higher levels in the hairy BDM group than in the hairless CYQ group (Figure 8B). Thus, high transcriptional levels of these DEGs have the potential to lead high accumulation of MCPs in the hairy tea cultivar. As trichomes protrude out, they likely experience more abrasion and wind-induced drying, and are usually covered with cuticular wax (Liang et al., 1993). Thus, we took the cuticular wax biosynthesis-related genes for further analysis. It is well established that cuticular wax biosynthesis begins with the elongation of long-chain fatty acyl precursors by the fatty acid elongase (FAE) multi-enzyme complex to the VLC fatty acyl-CoAs in epidermal plastids (Samuels et al., 2008). Then, these VLC fatty acyl-CoAs are exported to the endoplasmic reticulum, where they are catalyzed by cycles of four FAE complexes, including a KCS, a ketoacyl-CoA reductase (KCR), a HCD, and an enoyl-CoA reductase (ECR); finally forming the VLC acyl-CoAs with even total carbon number (Kunst and Samuels, 2009). In *Arabidopsis*, many genes encoding KCS and HCD were characterized by relatively high expression in trichomes compared to other epidermal cells (pavement cells and guard cells) (Hegebarth et al., 2016). In this study, RNA-seq results showed that *KCS* and *HCD* transcripts were abundant in the hairy BDM group (Figure 8B), suggesting that *KCS* and *HCD* may play pivotal roles in trichome cuticular wax development. In most plant species, two cuticular wax biosynthesis pathways (alkane- and alcohol-forming pathway) are generally involved in cuticular wax formation (Samuels et al., 2008). In the alkane-forming pathway, VLC acyl-CoAs resulting from FAE complexes elongation are reduced to aldehydes, which are decarbonylated to alkanes; CER1 and MAH1 are the key enzymes in this pathway (Kosma and Rowland, 2016). The researcher observed that trichome cuticular waxes have high alkane content, which is associated with relatively high *CER1* expression in *Arabidopsis* trichomes (Hegebarth et al., 2016). Of the three types of epidermal cells (pavement cells, guard cells, and trichomes), *MAH1* was expressed solely in developing trichomes (Hegebarth et al., 2016). Strikingly, the unigenes encoding CER1 and MAH1 were highly expressed in the hairy BDM group in the present study (Figure 8A). Also, in the alcohol-forming pathway, acyl-CoAs resulting from FAE complexes elongation are reduced to alcohols, which are esterified with free fatty acids to form wax esters; FAR and WSD1 are the vital enzymes in this pathway (Kosma and Rowland, 2016). In *Arabidopsis, CER4/FAR3* was expressed in trichomes but not in the pavement cells of rosette leaves (Rowland et al., 2006). In our study, two unigenes encoding FAR and their expressions remarkably increased in the hairy BDM group compared with the hairless CYQ group (Figure 8A). In addition, *WSD1* may also play an important role in trichome development. *WSD1* showed high expression levels in trichomes compared with other epidermis cells in *Arabidopsis* (Hegebarth et al., 2016). In the present study, DEGs encoding WSD1 were highly expressed in the hairy BDM group (Figure 8A). Additionally, *LACS* has also been implicated in plant trichome cuticular waxes development (Hegebarth et al., 2016). RNA-seq results suggested that six DEGs encoding LACS were remarkably upregulated in the hairy BDM group (Figure 8B). Taken together, the high transcriptional levels of cuticular wax-related genes in the hairy BDM group may play vital roles in the cuticular wax accumulation of tea plant trichomes. In addition, other cell wall-related genes such as *ELP, EXP*, and *PRP* may also play important roles in trichome development (Li et al., 2020b). In this study, we identified 20 DEGs encoding ELP, EXP, and PRP, that showed high expression levels in the hairy BDM group (Figure 8B). Furthermore, a unigene (TEA029695) encoding PRP displayed the positive expression trend with trichome length and width in the ME red module (Figure 11E). Collectively, our results suggested that cell wall materials biosynthesis-related key pathways and genes may be essential for trichome development in tea plants.

### Cell cycle and cytoskeleton structure may play critical roles in tea plant trichome initiation and formation

Plant trichome development is intimately related to cell cycle control (Yang and Ye, 2013). However, little attention has been paid to the expression of genes involved in the cell cycle process during tea plant trichome development. Thus, we systematically analyzed the key genes associated with the cell cycle pathway. Of the critical components during the cell cycle progression is the CDK enzyme (Inzé and De Veylder, 2006). In our work, we identified two *CDK2* genes; their transcriptional levels were significantly higher in the hairy BDM group than in the hairless CYQ group (Figure 9), suggesting that they may play important roles in the cell cycle pathway. It is known that cyclin, the essential activator of CDK, is required to coordinate cell division and differentiation during trichome development (Peeper et al., 1993). Tomato B-type cyclin, SlCycB2, plays a pivotal role in trichome initiation and development (Gao et al., 2017). *SlCycB2* was highly induced in the tomato *woolly* (trichome activator) mutant but suppressed in the *hairless* (trichome repressor) mutant (Yang et al., 2011; Tian et al., 2012). In this study, we found that the expression trend of *CycB* was closely related to tea plant trichome development (Figure 9), indicating that *CycB* may be essential for trichome initiation in a tea plant. Further, the transcriptional level of *CDC20*, critical for the cell cycle (Park et al., 2008), was significantly upregulated in the hairy BDM group than in the hairless CYQ group (Figure 9), indicating that *CDC20* may play a vital role in tea plant trichome formation. The roles of other cell cycle-related genes we identified were rarely reported in plant trichomes, which warrants further study.

The plant cytoskeletal system composes of microtubules (MTs) and actin filaments (F-actin), which play a cooperative role in regulating trichome cell morphogenesis (Chang et al., 2019). In *Arabidopsis*, F-actin mainly affects the elongation of trichomes, and MTs are responsible for trichome branching (Sambade et al., 2014). In our investigation, more DEGs in the BDM group annotated to the “cytoskeleton”-related GO terms compared to the CYQ group (Figures 4A,B). Further, we identified some cytoskeleton-related DEGs, such as *ACT, TUB, MAP, KIS*, and *MYO* (Figure 10). Among them, ACT protein is the major component of the plant cytoskeleton (Nakahama et al., 2018). In cotton, *GhACT1, GhACT2*, and *GhACT5* were involved in fiber elongation (Li et al., 2005). In *Arabidopsis, AtACT1* was required for trichome morphogenesis, and misexpression of *AtACT1* affected the normal branching and growth of trichomes (Kandasamy et al., 2002). In addition, TUB protein is a prominent component of microtubules (Nakahama et al., 2018). α*-tubulin 4* and α*-tubulin 6* play pivotal roles in *Arabidopsis* trichome branching (Tatsuya et al., 2004). The mutant α*-tubulin 6* allele made MTs stable and contributed to new branch formation (Abe and Hashimoto, 2010). Our WGCNA result showed that a *TUB* (TEA014466) and two *ACT* (TEA030184 and TEA005826) were the hub genes in the ME magenta and ME red modules, respectively (Figures 11C,E). Additionally, our RNA-seq results revealed that most unigenes encoding ACT and TUB were extensively expressed in the hairy BDM group compared with the hairless CYQ group (Figure 10), suggesting their roles in trichome development. Furthermore, ADF is a key actin-binding protein involved in the regulation of actin-cytoskeleton dynamics (Dong et al., 2001). In cotton, *GhADF1* played vital roles in regulating fiber elongation as well as cellulose deposition (Wang et al., 2019). In *Arabidopsis, AtADF9* was weakly expressed in nearly all vegetative tissues but was strongly expressed in trichomes (Burgos-Rivera et al., 2008). In addition, MAP engages MTs to regulate the MTs state and a wide variety of cytoskeletal functions (Li et al., 2017). In *Arabidopsis, MAP* family members (*WVD2* and *CLASP*) were involved in the regulation of trichome formation (Perrin et al., 2007; Zhu et al., 2018). In cotton, *GhCLASP2* regulated cotton fiber development, particularly cotton fiber strength, by interacting with MTs and affecting cellulose synthesis and deposition (Zhu et al., 2018). In this study, a *MAP* (TEA008191) was identified as the hub gene of the ME magenta module in the WGCNA result (Figure 11C), and the higher expression levels of most *ADF* and *MAP* genes in the hairy BDM group may suggest their roles in tea plant trichome development (Figure 10). Generally, cytoskeletal motor proteins are ATPases that use the energy released from ATP hydrolysis to move along the cytoskeletal elements of MTs and F-actin (Lee and Liu, 2004). Among all the eukaryotic organisms, KIS, a microtubule-based motor protein with the conserved kinesin motor domain, is responsible for ATP hydrolysis and microtubule binding (Lee and Liu, 2004). Previous studies found that some genes encoding KIS, such as *Kinesin-13A* and *ZWI*, were required for normal trichome morphogenesis in *Arabidopsis* leaves (Oppenheimer et al., 1997; Lu et al., 2005). GhKCH1, a kinesin isoform, was involved in cotton fiber cell growth *via* organizing microtubule array and actin network (Preuss et al., 2004). Meanwhile, MYO protein is an actin microfilament-based motor protein with the conserved myosin motor domain that uses the chemical energy stored in ATP for mechanical displacement (Lee and Liu, 2004). In *Arabidopsis*, a myosin protein, XIK was required for the coordinated expansion of trichomes branches and stalks (Ojangu et al., 2007). In another *Arabidopsis* study, three genes encoding MYO, *XI-K, XI-1*, and *XI-2* played vital roles in the expansion of trichomes, the development of root hairs, and the elongation of stigmatic papillae (Ojangu et al., 2012). Here, transcriptional levels of most *KIS* and *MYO* genes displayed relatively higher expression in the hairy BDM group compared with the hairless CYQ group (Figure 10). These results suggest that the cell cycle and cytoskeleton biosynthesis may be associated with tea plant trichome initiation and formation.

## Conclusions

Overall, we found a naturally hairy tea cultivar whose surface was covered by unicellular, unbranched, straight, and soft structured trichomes. Transcriptome analysis revealed that several DEGs, involved in TFs, PRGs, cell wall biosynthesis, epidermal cell cycle and division, and cytoskeleton structure possibly regulated trichome development in tea plants. This study will contribute to understanding tea plant trichome development, as well as provide some valuable candidate genes for molecular breeding in tea plants.

## Data availability statement

The original contributions presented in the study are publicly available. This data can be found at: NCBI, PRJNA858236.

## Author contributions

SL, ZL, JH, LC, and NT conceived and designed the work. LC, NT, MHu, DS, QJ, and MG performed the experiments. LC, SL, XZ, YP, JZ, ZC, GL, and MHua analyzed the data. LC and NT wrote the paper. LC, SL, and DS revised the paper. All authors have read and approved the manuscript.

## Funding

This work was financially supported by the National Key Research and Development Program of China (No. 2021YFD1200200), National Natural Science Foundation of China (32172629, U19A2030, and 31670689), Provincial Natural Science Foundation of Hunan (2020JJ4358), Hunan Provincial Seed Industry Innovation Project (2021NK1008), and Open-end Fund of Key Laboratory of Biology, Genetics and Breeding of Special Economic Animals and Plants, Ministry of Agriculture and Rural Affairs (TZDZW202207).

## Conflict of interest

The authors declare that the research was conducted in the absence of any commercial or financial relationships that could be construed as a potential conflict of interest.

## Publisher's note

All claims expressed in this article are solely those of the authors and do not necessarily represent those of their affiliated organizations, or those of the publisher, the editors and the reviewers. Any product that may be evaluated in this article, or claim that may be made by its manufacturer, is not guaranteed or endorsed by the publisher.
